# Optimal Contribution Selection Improves the Rate of Genetic Gain in Grain Yield and Yield Stability in Spring Canola in Australia and Canada

**DOI:** 10.3390/plants12020383

**Published:** 2023-01-13

**Authors:** Wallace A. Cowling, Felipe A. Castro-Urrea, Katia T. Stefanova, Li Li, Robert G. Banks, Renu Saradadevi, Olaf Sass, Brian P. Kinghorn, Kadambot H. M. Siddique

**Affiliations:** 1 The UWA Institute of Agriculture, The University of Western Australia, Perth, WA 6009, Australia; 2 UWA School of Agriculture and Environment, The University of Western Australia, Perth, WA 6009, Australia; 3 Animal Genetics and Breeding Unit, University of New England, Armidale, NSW 2351, Australia; 4 Norddeutsche Pflanzenzucht Hans-Georg Lembke KG, Hohenlieth, 24363 Holtsee, Germany; 5 School of Environmental and Rural Science, University of New England, Armidale, NSW 2351, Australia

**Keywords:** genetic gain, overall performance, grain yield, yield stability, canola, rapeseed, *Brassica napus*, pedigree BLUP, estimated breeding values, optimal contributions selection

## Abstract

Crop breeding must achieve higher rates of genetic gain in grain yield (GY) and yield stability to meet future food demands in a changing climate. Optimal contributions selection (OCS) based on an index of key economic traits should increase the rate of genetic gain while minimising population inbreeding. Here we apply OCS in a global spring oilseed rape (canola) breeding program during three cycles of S_0,1_ family selection in 2016, 2018, and 2020, with several field trials per cycle in Australia and Canada. Economic weights in the index promoted high GY, seed oil, protein in meal, and Phoma stem canker (blackleg) disease resistance while maintaining plant height, flowering time, oleic acid, and seed size and decreasing glucosinolate content. After factor analytic modelling of the genotype-by-environment interaction for the additive effects, the linear rate of genetic gain in GY across cycles was 0.059 or 0.087 t ha^−1^ y^−1^ (2.9% or 4.3% y^−1^) based on genotype scores for the first factor (f_1_) expressed in trait units or average predicted breeding values across environments, respectively. Both GY and yield stability, defined as the root-mean-square deviation from the regression line associated with f_1_, were predicted to improve in the next cycle with a low achieved mean parental coancestry (0.087). These methods achieved rapid genetic gain in GY and other traits and are predicted to improve yield stability across global spring canola environments.

## 1. Introduction

The rate of gain in grain yield (GY) in the world’s major grain crops ranges from 0.9 to 1.6% y^−1^ and should at least double to meet the expected global demand for grain crops in 2050 [1,2,3]. In some regions, GY is stagnating or declining due to climate change [2,4]. Plant breeders face a major challenge to accelerate genetic gain in GY during the forthcoming period of global population growth and climate change [1,5], and new methods of rapid breeding should involve wide-scale phenotyping, accurate selection, and international exchange of elite varieties [6]. 

One potential method of accelerating genetic gain in self-pollinating crops is the use of rapid cycles of early-generation recurrent selection [7]. Such methods were attempted previously and were successful for one or a few traits [8] but fell out of favour due to their lack of application to commercial crop breeding, where multiple traits were under selection. The aim of this study was to field-test stochastic models of early-generation recurrent selection in a self-pollinating crop [9,10], which showed great promise for high genetic gain in multiple economic traits. This field validation of genetic gain occurred within a commercial spring oilseed rape (canola) breeding program.

Genetic gain in plant breeding programs can be assessed by a variety of methods [11] and the most common method is the evaluation of released varieties in historical variety trials [1]. Genetic gains in crop breeding programs have been estimated in stochastic models [9,10,12] or in *ex post facto* analysis of recurrent selection programs in crops such as maize, oats, barley and rice [5,11,12], but rarely are genetic trends measured within active crop breeding programs.

Assessment of genetic gain may be based on linear mixed models [13,14], but these are subject to bias in estimating genetic and non-genetic (environmental) trends [15]. Bias can be avoided by fitting fixed regression terms for genetic and non-genetic trends [16]. The predicted breeding value (PBV) of an individual is influenced by the environment in which the phenotype is measured, and a trend in the environment over time may be confounded with a trend in PBV [17]. Measures on relatives in highly connected ancestral pedigrees help to avoid this possibility. Garrick [18] used multiplicative mixed models (MMM) to partition phenotypic trends into genetic and environmental components. If the genetic trend and environmental trends are in opposite directions, or the genetic trend persists after incorporating a year effect, this supports the existence of a genetic trend over years [19,20]. 

Variety-by-environment interaction effects will also influence genetic gain over time and may be accounted for in MMM with factor analytic (FA) modelling of the variety-by-environment effects. Stefanova and Buirchell [21] used MMM-FA analysis of historical variety trials of GY in narrow-leafed lupins and showed that the variety scores for the first factor (f_1_) of an FA(2) model were a measure of variety performance and scores for the second factor (f_2_) were a measure of variety stability across the environments. Scores for f_1_ and f_2_ were expressed in trait units in Figures 3 and 4 of Stefanova and Buirchell [21]. The rate of genetic gain in GY over 31 years from the release of ‘Unicrop’ to ‘Mandelup’ was 17 kg ha^−1^ y^−1^ based on f_1_ scores expressed in trait units in Figure 4 of Stefanova and Buirchell [21]. However, when based on the average variety predicted means across environments, the estimate increased to 20 kg ha^−1^ y^−1^ in Figure 3 of Stefanova and Buirchell [21]. 

Smith and Cullis [22] used a similar approach to measure overall performance (OP) across environments, which was based on f_1_ scores in MMM-FA models expressed in trait units. OP was equivalent to a generalised main effect of variety performance when the scale differed across environments [22]. Variety stability, also expressed in trait units, was the root-mean-square deviation (RMSD) from the regression line associated with f_1_. OP and RMSD were deemed to be the two main drivers in the selection process [22]. 

In this study, we compare two methods of estimating rates of genetic gain for GY and other traits in an actively evolving spring oilseed rape breeding program based on MMM-FA models: the average predicted breeding values (PBV) across environments, and OP. For the first time in crop breeding, we use RMSD as a measure of yield stability in a selection index. Our estimate of genetic trends across cycles is based on an individual model (or animal model) and depends on sufficient depth and connectedness of pedigrees across cycles [20]. We accumulate data across cycles and estimate heritability and genetic correlation between traits at the end of each cycle. In previous studies, the average accuracy of breeding values for a low heritability trait was high (*r* > 0.80) in non-inbred lines based on an individual model [23].

Knowledge of current genetic trends in the breeding program allows timely decisions to be made to optimise the program, such as rapid responses to changes in technology, markets, or the environment [24]. For example, the weighting on heat stress tolerance among other traits such as GY and disease resistance in the economic index can be adjusted to meet future anticipated increases in global temperatures [10].

Optimal contributions selection (OCS) was first used in self-pollinating crops in stochastic models of the additive effects in rapid cycles of early generation selection [9,10,25]. OCS in MateSel [26] includes a crossing plan which optimises parental contributions and balances genetic gain for the index under a number of possible constraints or weightings on factors that affect genetic diversity, inbreeding rate and inbreeding level [27,28]. Importantly, MateSel permits the breeder to predict the genetic gain for each trait and achieved parental coancestry in the next cycle. This allows for timely adjustments in the breeding program for changes in future desired gains, such as adjustments to weightings on traits in the economic index or changes to selection targets.

In S_0,1_ family selection, the S_0_-derived S_1_ family provides an estimate of the breeding value of the S_0_ individual for selection purposes [20]. In stochastic models, rapid cycles of S_0,1_ family selection (two years per cycle) with OCS and optimised mating designs improved the rate of long-term genetic gain and reduced the rate of population inbreeding compared to truncation selection and random mating among selected parents [10]. For practical reasons, the S_0,1_ family seeds are grown in plots in field trials, and the agronomic and harvested grain traits recorded on these plots are used to predict the breeding value of the S_0_ individuals. Accurate PBV (*r* > 0.80) were generated on S_0_ individuals for GY, disease resistance, agronomic traits, and seed quality in stochastic models of S_0,1_ family selection based on a deep and highly interconnected pedigree [9,10]. 

**Figure 1:**
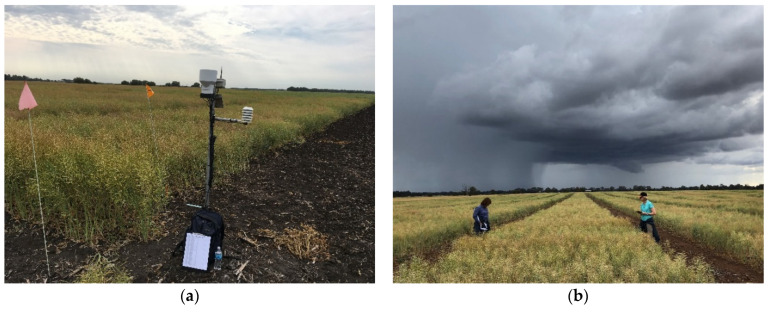
Concurrent 2018 field trials of S_0,1_ families of spring canola located (**a**) in Canada (trial code 2018CA1 at Sun Valley, Manitoba, Canada) and (**b**) in Australia (trial code 2018AU2 trial at Rutherglen, Victoria, Australia). For trial codes, see Table 1.

In this field study, depending on seed availability, each S_0,1_ family was grown in replicated plots at multiple sites and in multiple regions; we grew concurrent field trials of the same S_0,1_ families in Australia and Canada (Figure 1). We included data for GY and several major economic traits including flowering time, plant height, seed quality traits, and resistance to Phoma stem canker or blackleg disease caused by *Leptosphaeria maculans*. Resistance to blackleg disease is important for secure production of oilseed rape in Australia, Canada, and Europe [29,30].

Augmented S_0,1_ family selection exploits complex relationships between genotypes within and between cycles [10] to generate accurate PBV in an individual model analysis with phenotypic and relationship data from all cycles of selection [17]. As a side bonus, the method also results in near-homozygous lines after two or three cycles that are ready for commercial evaluation [10]. Genomic relationship information can be added at any time and combined with pedigree relationship information in single-step genomic prediction [31]. New germplasm can be added to the program in any cycle. In some crops, it may be necessary to bulk seeds from single S_0_ plants over 2 selfing generations (S_0,2_ bulks) to generate sufficient seed for phenotyping [20], such as in common bean [32].

In this study, we use MMM-FA analysis of multi-environment trials across cycles to assess the rate of genetic gain for GY and several economic traits inside an active global spring oilseed rape (canola) breeding program during three cycles of augmented S_0,1_ family selection. We compare the rate of genetic gain in GY across cycles by two methods, OP and average PBV across environments. For the first time in crop breeding, we include yield stability across global environments based on RMSD in the economic index. This study also represents the first major attempt to integrate spring oilseed rape germplasm across the southern and northern hemispheres.

## 2. Materials and Methods

### 2.1. Terminology

**Figure 2:**
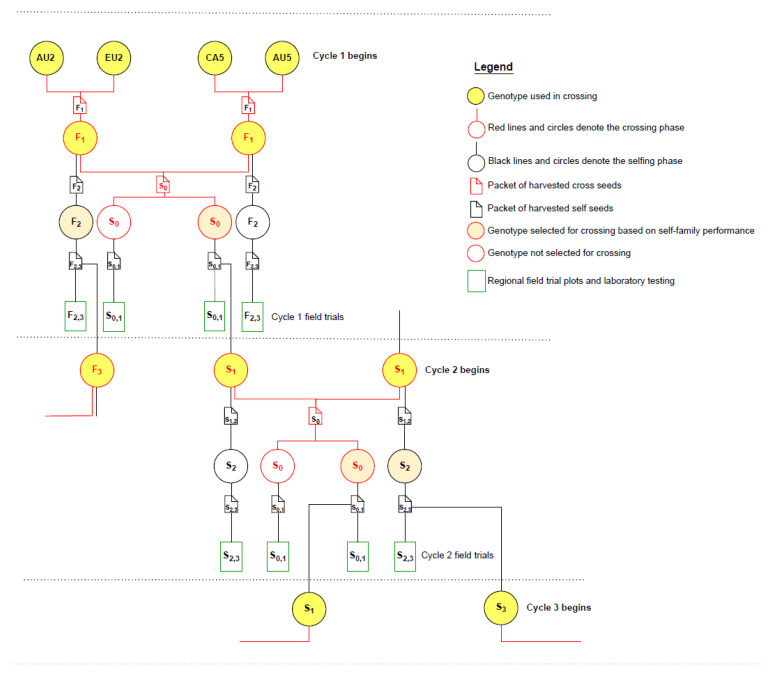
Two cycles of augmented S_0,1_ family selection showing genotypes in the pedigree. In cycle 1, inbred founder parents from Australia (AU) were crossed with parents from Europe (EU) or Canada (CA), and their F_1_ progeny were intercrossed to generate S_0_ and F_2_ progeny (circles). Individual S_0_ and F_2_ progeny were selfed and the self-seed was sown in S_0,1_ and F_2,3_ plots in cycle 1 field trials (rectangles). Phenotypic data from S_0,1_ and F_2,3_ plots in cycle 1 field trials were used to calculate the predicted breeding value of S_0_ and F_2_ progeny. Crossing in cycle 2 was based on remnant S_0,1_ or F_2,3_ seed of superior S_0_ and F_2_ progeny. A similar process occurred in cycle 2, with phenotypic data from S_0,1_ and S_2,3_ plots used to assess the predicted breeding value of S_0_ and S_2_ progeny genotypes, respectively. From cycle 2 onwards, selection was based on an economic index composed of all traits, and mating designs were derived from optimal contributions selection.

We use the traditional definitions in crop breeding of ‘F_1_’ as progeny of crosses between near-homozygous founder varieties, and ‘S_0_’ as progeny of crosses between heterozygous parent plants. F_1_ progeny are heterozygous and genetically uniform, and segregation occurs in the F_2_ generation after self-pollination. S_0_ progeny are non-inbred and therefore heterozygous and heterogeneous, that is, each S_0_ progeny is a unique genotype and segregation occurs in the S_0_ for multiple traits. Each plant in the ancestral pedigree is a unique genotype and has a relationship with every other genotype in the pedigree, whether the genotype is derived from crossing or selfing (Figure 2). S_0,1_ represents the selfed progeny from an S_0_ plant [20].

In this study, the term ‘cycle’ refers to a cycle of recurrent selection, that is, the generation interval (L) as used in the breeder’s equation [20]. One cycle in this study takes two years, which includes the time taken to cross the parent plants, self the S_0_ to generate S_0,1_ families, phenotype the S_0,1_ families in the field trials and the laboratory, and select parents for crossing to begin the new cycle (Figure 2). One or more selfing generations may occur within a cycle. In this study, S_0,1_ family selection [20] is augmented by taking forwards S_2_ self progeny of parent plants in addition to S_0_ cross progeny for phenotyping in the next cycle (in field plots of S_2,3_ or S_0,1_ families), and this increases the number of collateral relatives and pedigree linkages across cycles of selection (Figure 2). 

### 2.2. Founder Population, Crossing and Selfing to Begin Cycle 1

The founder population included 32 southern hemisphere (SH) elite spring canola lines from Australia (AU1 to AU32) and 32 northern hemisphere (NH) elite spring canola lines from Canada (CA1 to CA16) and Europe (EU1 to EU16) which were intercrossed in 32 pair-wise combinations (SH × NH) in 2012. Crossing occurred in glasshouses at The University of Western Australia Field Station, Shenton Park, Western Australia. Their F_1_ progeny were intercrossed from January to June 2013 in 16 combinations (F_1_ × F_1_). For example, the F_1_ of AU2 × EU2 was intercrossed with the F_1_ of CA5 × AU5 to generate S_0_ progeny D1 (Figure 2, Online Resource S1). All female parents were selected for triazine tolerance and therefore the breeding population was uniformly tolerant of triazine herbicides due to the cytoplasmic control of this trait [33]. This avoided any potential bias in the project stemming from segregation among progeny for herbicide tolerance. 

In cycle 1, S_0_ and F_2_ progeny were grown in the field in Chile from October 2013 to February 2014 where selfing occurred inside pollination bags on single plants. S_0,1_ and F_2,3_ families were returned to Australia where they were sown in the cycle 1 field trial in May 2014 (Figure 2). Recurrent selection cycles continued every two years and 70 new migrants (35 from SH and 35 from NH) were added during cycles 2, 3 and 4 as F_1_ progeny of the crosses SH × NH (Online Resource S2).

### 2.3. Cycles of Augmented S_0,1_ Family Selection

The cycle 1 field trial (trial code 2014AU1) was grown in Western Australia from May to November 2014 and included 668 S_0,1_ families derived from F_1_ × F_1_ matings augmented with 577 F_2,3_ families derived from F_2_ seed harvested from each F_1_ parent plant (Table 1). In addition, several S_1,2_ and F_3,4_ families were evaluated in 2014 (Table 1). Remnant seeds of S_0,1_, F_2,3_, S_1,2,_ and F_3,4_ families were stored for potential future use as parents to begin cycle 2. Crossing to begin cycle 2 was among selections from cycle 1 progeny which performed well for GY and grain quality traits in trial 2014AU1, and 170 matings were made by the breeder to combine different pedigrees in male and female parents. Crossing decisions to begin cycles 3, 4, and 5 were based on optimal contributions selection (OCS) as described below in Section 2.8 ‘Optimal Contributions Selection and Crossing’.

The process of augmented S_0,1_ family selection continued in two-year cycles with phenotyping of S_0,1_ and S_2,3_ families in multiple field trials in Australia and Canada in cycles 2 (2016), 3 (2018) and 4 (2020) (Table 1, Online Resource S2). There were two field trials in cycle 2, three in cycle 3, and three in cycle 4. Trials in Canada were located at Sun Valley, Manitoba (trial codes 2018CA1 and 2020CA1), and in Australia were located in canola cropping zones near Perth, Western Australia (trial codes 2014AU1, 2016AU1, 2018AU1, and 2020AU1) and in northern Victoria (trial codes 2016AU2, 2018AU2 and 2020AU2). Sites identified as AU2 were ‘disease nurseries’ sown on straw of the previous year’s canola crop to promote high levels of Phoma stem canker (blackleg) disease (Table 1a). Connectivity of genotypes, that is, the occurrence of the same genotypes in different trials, was high across trials within cycles but low across trials between cycles (Table 1b).

### 2.4. Pedigree Structure and Relationships 

As required for pedigree analysis in ASReml-R v4 [34], each genotype was described in the pedigree file with the name of the genotype, the names of its male and female parents and its level of selfing (‘fgen’). This was preceded in the file by rows which showed the same information for each parent. The pedigree included ancestors of founder varieties and control varieties. For most genotypes, fgen was the default value of zero because selfing was explicitly described in the pedigree file (the male and female parents were identical). For most inbred ancestral varieties fgen was set at 5 and for those derived from doubled haploids fgen was set at 10. The pedigree file was converted into an additive genetic relationship matrix and an inverse relationship matrix was formed for analysis in ASReml-R v4 [34]. The pedigree was highly interconnected within and across four cycles as a result of selfing and crossing in augmented S_0,1_ family selection (Figure 2, Online Resource S1). 

Inbreeding coefficients (F) (Online Resource S3) and coefficients of coancestry (f) (Online Resource S4) were calculated from the additive genetic relationship matrix (A-matrix). F was calculated as the A-value on the diagonal of the A-matrix minus 1, and f was calculated as ½ the A-value of the 2 parent genotypes.

### 2.5. Field Trials and Phenotyping

S_0_ and S_2_ genotypes were evaluated as S_0,1_ or S_2,3_ families in field plots (Figure 2) with one or two replicates per site and at multiple trial sites per cycle depending on seed availability. Phenotypes of the S_0,1_ or S_2,3_ families were used to predict the breeding value of the S_0_ and S_2_ individuals for selection purposes [20], as described below in Section 2.6 ‘Data Analysis’. Historical triazine tolerant cultivars were also included in some trials as controls with one or two replicates per site, with name and year of release: Karoo (1996), ATR Beacon (2002), ATR Stubby (2003), Bravo TT (2004), Tornado TT (2004), Banjo TT (2005), Crusher TT (2010), ATR Gem (2011), ATR Stingray (2011), Sturt TT (2012), ATR Wahoo (2013), ATR Bonito (2013), and ATR Mako (2015) [35].

Partially replicated field trials [36] were designed in DiGGer (available from http://nswdpibiom.org/austatgen/software/, accessed 9 January 2023) with the default spatial model at sites in Australia and Canada (Table 1). Each site was grown under natural rainfall with date of sowing and agronomic treatments similar to commercial canola production in the region. Each field plot was 6-m long and six rows wide (1.8 m centre-to-centre) and sown with 3 g seed per plot. Plots were cut back to 4 m prior to harvest. Control cultivars and S_0,1_ and S_2,3_ families with sufficient seed were replicated at each site (Table 1). The proportion of plots at a site composed of single-replicate genotypes varied from 37.7% to 83.9% over the four cycles (Table 1).

**Figure 3:**
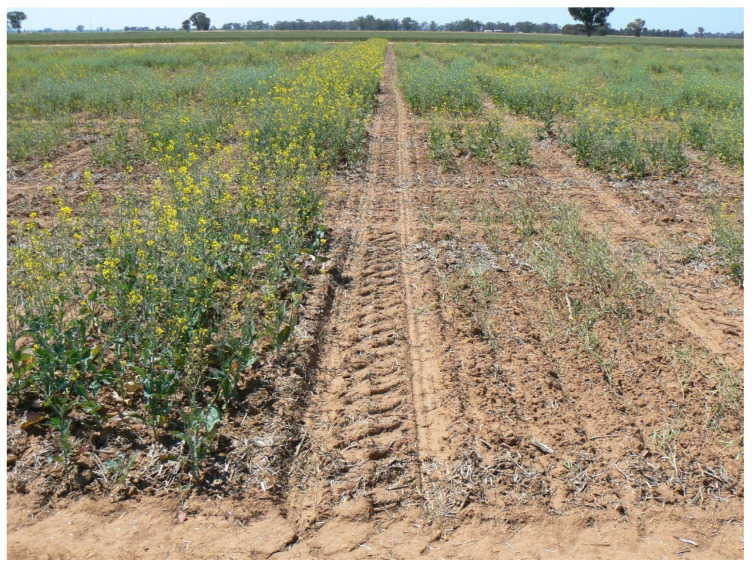
Impact of blackleg (Phoma stem canker) disease in the disease nursery field trial 2016AU2 at the late flowering stage. The 4-m × 1.8-m plot on the left was intermediate in resistance (BL score 5) and the plot on the right was very susceptible (BL score 1).

During the growing season at some sites, plots were scored for days to 50% flowering (DTF) and plant height at maturity (PlHt, cm). At disease nursery sites (AU2), plots were also scored for blackleg resistance at maturity. The blackleg (BL) disease resistance score was based on the number of plants that were visibly dead or lodging as a result of blackleg disease in the plot at pod filling stage, and BL scores ranged from 1 (very susceptible, no plants survived blackleg disease) to 9 (very resistant, all plants survived) (Figure 3). Two assessors scored each plot and the average of their scores was recorded as the BL score on the plot. 

Field trial plots were harvested by small plot harvester and the weight of harvested seed per plot was converted to grain yield per hectare (GY, t ha^−1^). Harvested grain from each plot was stored in a dry environment and one hundred seeds were randomly extracted from harvest bags to measure 100 seed weight (SW100, g).

Samples of harvested seed from each plot (5 g) were assessed by near-infrared radiation spectroscopy for moisture content (%), seed oil (Oil, % of seed, adjusted to 6% moisture), protein in meal (ProM, % of meal, adjusted to 10% moisture), glucosinolates (GSL, μmole g^−1^), and oleic acid (OL, % of total fatty acids) after pre-calibration against standard seed samples with known levels of moisture, Oil, ProM, GLS, and OL [37].

### 2.6. Data Analysis

The statistical models follow those of previous authors [21,38,39,40,41,42] as shown in Online Resource S5 and explained below.

#### 2.6.1. Preliminary Single Site Analysis 

Univariate single-site analyses were conducted in ASReml-R v4 [34] which produces residual maximum likelihood (REML) estimates of the variance parameters [43] and best linear unbiased predictions (BLUP) [44] of the random effects. Univariate analyses were general linear mixed models which followed the structural specifications presented in Gilmour et al. [45] (Online Resource S5).

The spatial variation of each trait within each trial was assessed following the mixed model approach described by Gilmour et al. [46] and Stefanova et al. [47]. Trends along rows and columns were assessed in an autoregressive residual model (AR1 × AR1) and significant linear row and column trends and/or random row and column effects were fitted to the model following Stefanova and Buirchell [21] and Beeck et al. [38]. The effects of local spatial trends were assessed using a plot of residuals, the sample variogram, row and column faces of the empirical variogram, and REML likelihood ratio tests (LRT) [47].

‘Genotype’ was considered a random genetic effect since large numbers of progeny genotypes were randomly assigned to field trials, there was no prior selection by the breeder, and the goal was to rank progeny genotypes for relative performance. Random effects were assessed for significance by the Z-test of variance components and fixed effects were assessed by the significance of the Wald statistic. 

Finally, the additive genetic relationship matrix was added to the model to estimate the additive and nonadditive genetic effects (Online Resource S5). The subsequent improvement in the model was assessed by REML LRT, Akaike information criterion (AIC) [48], and Bayesian information criterion (BIC) [49]. Possible outliers were assessed and removed. 

#### 2.6.2. Base Univariate Model Across-Sites 

The base model univariate analysis across-sites was a MMM and included the additive genetic relationship matrix and significant fixed and random terms from the single site analysis but ignored covariances between sites, following Stefanova and Buirchell [21] and Beeck et al. [38]. ‘Site’ was considered a fixed effect. The main effect of ‘Genotype’ was excluded and ‘Genotype × Site’ was included as a random effect since the goal was to rank genotypes for relative performance at each site (Online Resource S5). Significant spatial terms from the single-site analyses were transferred to the base model. Variance components were estimated for additive and nonadditive genetic effects at each site and narrow-sense heritability for each trait at each site was calculated as the ratio of the additive variance component divided by the sum of additive, nonadditive, and error variance components. The average accuracy of the additive effects ($r_{i}$) was calculated for the i-th individual for each trait at each site, following Gilmour et al. [45] for the animal model. 

#### 2.6.3. Factor Analytic Modelling of Each Trait across Sites

Across-sites analysis of each trait proceeded with an MMM-FA model (Online Resource S5) following the approach in Stefanova and Buirchell [21] and Beeck et al. [38] where ‘Site’ was considered a fixed effect and ‘Genotype × Site’ a random effect. MMM-FA included the additive genetic relationship matrix and significant fixed and random spatial terms from the base model. Both additive and non-additive genetic effects were assessed in the MMM-FA when significant [38]. Analysis proceeded sequentially in ASReml-R from the base model to first-order factor FA(1), second-order factor FA(2), and so on until the optimum MMM-FA model was chosen based on a non-significant or trivial improvement in the REML LRT, AIC and BIC tests or percentage variance accounted for in the next higher order model. 

The PBV for an individual was calculated by averaging the predicted variety means across environments based on the optimum MMM-FA model [21]. 

Genetic correlations of additive effects of genotypes across sites were obtained from the optimum MMM-FA model.

#### 2.6.4. Genotype Overall Performance and Stability 

The average PBV for an individual across environments was a measure of genotype performance across environments based on the optimum MMM-FA model, following the approach for variety predicted means across environments [21]. A second measure of genotype performance across environments was overall performance (OP) which was based on f_1_ scores in the optimum MMM-FA model and expressed in trait units. OP was deemed to be a generalised main effect of variety performance when scale differed across environments [22]. 

We compared two measures of stability of genotype performance across environments: scores for the f_2_ from the optimum MMM-FA model [21] and the root-mean-square deviation (RMSD) from the regression line associated with f_1_ in the optimum MMM-FA model [22]. 

### 2.7. Economic Index

The PBV for each trait from the optimum MMM-FA model for each genotype was weighted by an economic value and summed across traits to form an economic index for each genotype in the ancestral pedigree. An example of a hypothetical genotype is provided in Table 2.

Firstly, the total GY of each genotype was calculated as the sum of the PBV GY of the genotype and population mean GY, expressed in t ha^−1^. The total GY of the genotype was multiplied by the average contemporary market value of harvested grain in US$ t^−1^ to find the contribution of the GY of the genotype to the economic index in US$ ha^−1^ (Table 2).

The economic weight for Oil was based on the bonification rate for oil in Australia where there is a 1.5% grain price premium (or deduction) for each 1% seed oil above (or below) the base seed oil content of 42% [50]. The economic weight for +1 unit PBV Oil is converted into US$ t^−1^ (US$8.25 t^−1^) based on grain price US$550 t^−1^. The contribution of Oil to the economic index in US$ ha^−1^ was calculated as the PBV Oil of the genotype × economic weight for +1% Oil (US$8.25 t^−1^) × total GY of the genotype (2.343 t ha^−1^) (Table 2).

The economic weights on ProM, DTF, PlHt, GSL, OL, SW100, and BL in the economic index were subjectively assigned and modified to achieve the desired outcomes in the next cycle of the optimised mating design. Positive weights were assigned for traits where genetic gains were desired upwards (ProM, BL, SW100), and negative weights where genetic gains downwards were desired (DTF, PlHt, GSL), or zero weights where no change was desired (OL) (Table 2).

### 2.8. Optimal Contributions Selection and Crossing

The inter-crossing of founders to begin cycle 1 is described above in Section 2.2 ‘Founder Population, Crossing and Selfing to Begin Cycle 1’. Crossing to begin cycle 2 was among selections from cycle 1 progeny which performed well for GY and grain quality traits in trial 2014AU1, and 170 matings were made by the breeder with an emphasis to combine different pedigrees in male and female parents (Online Resource S2).

Crossing designs for cycles 3, 4, and 5 were based on OCS in the implementation platform ‘MateSel’ for the construction of an optimised mating design [26]. The selection and mate allocation method used previously [9,10,28] involves a function whose key components relate to genetic gain and genetic diversity. The optimisation of this function results in OCS. The practical implementation of this method is based on an evolutionary algorithm, with weightings and constraints easily invoked to ensure practical relevance, precise control of the response to each trait in the economic index, and other requirements of progressive breeding programs. MateSel dictates which individuals to select and the actual mating allocations and/or selfings to be made, based on the breeder’s identification of candidates for crossing and the maximum permissible number of crosses for each candidate.

MateSel was instructed to design 250 matings (to initiate cycles 3 and 4) and 150 matings (to initiate cycle 5) (Online Resource S2) from candidates based on a conservative balance strategy of target 45 degrees or higher, where 0 degrees gives full emphasis to short term genetic gain in index and 90 degrees gives full emphasis to minimizing parental coancestry [26]. Each genotype was limited to a maximum of 30 matings which was normally the maximum number of remnant self seeds. Remnant self seeds of selected genotypes were sown in the glasshouse as parent plants for crossing, based on the mating design derived from MateSel. 

### 2.9. Assessment of Genetic Gain 

Two types of genetic gain were assessed in this study — genetic gain in the breeding population over cycles, and genetic gain in historical cultivars included as controls in the trials over year of release. 

The rate of genetic gain in the breeding population was estimated from the linear regression over cycles of PBV or OP of candidate genotypes in cycles 2, 3, and 4 from the optimum MMM-FA analysis which included all data from cycles 2, 3, and 4. 

The rate of genetic gain in Australian historical cultivars was assessed by regressing the PBV or OP of historical cultivars based on the optimum MMM-FA analysis over their year of release. The Australian historical cultivars are listed in Section 2.5 ‘Field Trials and Phenotyping’ (above).

## 3. Results

### 3.1. Environmental Trends over Cycles and Countries

On average, 0.6% of plots at each trial site were invalid for various reasons and were excluded from the analysis of GY (Online Resource S6). 

**Figure 4:**
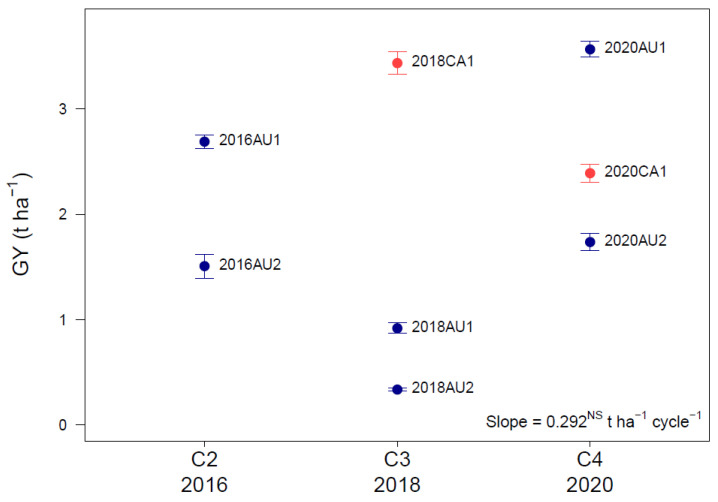
Lack of an environmental trend in site predicted grain yield (GY, t ha^−1^) ± standard error in field trials in Australia and Canada across cycles 2 (C2 2016), 3 (C3 2018) and 4 (C4 2020) from the optimum MMM-FA model of the genotype by environment effects (see Table 2). The slope of the linear regression of the predicted site mean GY across cycles was not significant (NS). For a description of trial codes, see Table 1.

The predicted site mean GY from the MMM-FA base model varied in Australia and Canada from site to site and cycle to cycle with no significant environmental trend in GY over cycles (Figure 4, Online Resource S6). Mean GY across cycles 2, 3, and 4 was 2.02 t ha^−1^ and fluctuated from 0.33 t ha^−1^ (trial 2018AU2, affected by late-season drought) to 3.57 t ha^−1^ (trial 2020AU1) (Figure 4, Online Resource S6). 

The average DTF was 104.1 days in Australia and 44.3 days in Canada, which reflects the different responses of spring canola to the winter-spring growing season in Australia and the summer growing season in Canada (Online Resources S6, S7). Following this trend, the average PlHt was higher in Australia (129.2 cm) than in Canada (103.1 cm) (Online Resources S6, S7). Average Oil was 46.9% in Australia and 40.4% in Canada, in contrast to average ProM which was 39.8% in Australia and 43.6% in Canada. The average GSL was 11.3 μmole g^−1^, the average OL was 61.7%, the average SW100 was 0.325 g and the average BL score was 5.1 (Online Resources S6, S7). There were no significant environmental trends over cycles in site mean values of any trait except a very small upward trend in SW100 (Online Resources S6, S7).

### 3.2. Genetic Relationships of Genotypes within and across Cycles

#### 3.2.1. Connectivity of Genotypes within and across Cycles

The connectivity of genotypes between trials within cycles was high (each S_0,1_ bulk was tested at multiple sites within cycles) but low across cycles (between 1 and 9 control cultivars were common across cycles) (Table 1b). However, the connectivity of the pedigree across cycles was high as a result of the use of both cross-and-self-sibs, that is, S_2_ self-progeny (evaluated as S_2,3_ families) and S_0_ cross progeny (evaluated as S_0,1_ families), and this increased the accuracy of additive genetic values in the base model (Online Resource S6). A small portion of the pedigree shows this high level of connectivity between cycles as a result of selfing and crossing of parent plants (Online Resource S1). The connectivity of genotypes between cycle 1 and the subsequent cycles was very low (Table 1b), and hence this cycle was excluded from further analysis.

#### 3.2.2. Inbreeding Coefficients and Coefficients of Coancestry 

The inbreeding coefficient (F) of individuals in the pedigree increased as the level of selfing increased and the coefficient of coancestry (f) between genotypes also increased as selfing increased in parent genotypes. For example, the F-values of individuals A1 and A2 in the pedigree diagram (Online Resource S1) were 0.0 and 0.5, respectively, as expected for F_1_ and F_2_ progeny of elite homozygous founder lines AU1 × EU1 (Online Resource S3). Founder lines AU1 and EU1 are assumed to be unrelated because the breeding programs in the southern hemisphere and northern hemisphere were mostly genetically isolated prior to 2000 [51]. Cycle 1 genotype C1 is an S_0_ progeny of the cross between two F_1_ genotypes A1 × B with F-value 0.019 (Online Resource S3), which reflects a small level of inbreeding between the two Australian founder lines AU1 and AU3 or between the European founder lines EU1 and EU3 prior to their use as founder parents. Self-progeny of C1 increased in F-value as the level of selfing increased from 0.509 in S_1_ genotype C2, 0.755 in S_2_ genotype C3, through to 0.969 in S_5_ genotype C6 (Online Resources S1, S3). 

The F-values in S_0_ cross progeny varied greatly between crosses depending on the level of common ancestors in the parents, but independently of the F-values in the parent genotypes. Cycle 4 S_0_ genotype Q (progeny of cross J4 × O3) had an F-value 0.326 due to several ancestors in common between parents J4 and O3 prior to cycle 4, but cycle 4 S_0_ genotype P (progeny of cross C6 × O2) had a much lower F-value 0.077 as a result of very few common ancestors between parents C6 and O2, despite the fact that one parent C6 had a very high F-value of 0.969 (Online Resources S1, S3).

The coefficient of coancestry (f) among full-sib progeny increased as the selfing level in parent genotypes increased. For example, the f-value of cycle 1 full-sib progeny G1 and H1 was 0.262 (Online Resource S4), which is close to the expected value of 0.25 because the parent plants were F_1_ genotypes and not inbred and not related. The f-value is slightly elevated due to a small level of inbreeding between the two Australian founder lines AU5 and AU6 or between the Canadian founder lines CA5 and CA6 prior to their use as founder parents (Online Resources S1, S4). However, the f-value of full-sibs J1 and K in Cycle 2 was higher than expected at 0.389 due to partial inbreeding in their S_1_ parents E2 and H2 (Online Resource S4). 

J1 is a cross progeny and E3 is a self-progeny of their common parent, E2, and J1 and E3 are cross-and-self-sibs with an expected f-value equivalent to full-sibs. The actual f-value between J1 and E3 was 0.388 due to partial inbreeding in their common S_1_ parent E2 (Online Resources S2, S4). 

Coefficients of coancestry between offspring of different parents in the population varied greatly. The f-value between cycle 4 S_0_ genotypes P and Q (0.262) was relatively high due to the presence of several common ancestors (especially O1 and J1), but the f-value between cycle 4 S_0_ genotypes J1 and I (0.080) was low since they shared no recent common ancestors (Online Resources S2, S4).

In summary, coefficients of coancestry between genotypes were often higher than expected due to selfing in parent genotypes, and this was helpful to increase the accuracy of PBV. Likewise, the presence of cross-and-self-sibs in addition to full-sibs and many other collateral relatives helped to increase the accuracy of additive genetic values (Online Resource S6) and increased pedigree connections across cycles. High coefficients of coancestry induced by selfing within cycles did not necessarily contribute to population inbreeding because optimized crossing designs favoured matings between unrelated genotypes with OCS in MateSel [26].

### 3.3. Analysis of Sites and Cycles

#### 3.3.1. Base Across-Sites Model 

Significant spatial trends fitted in the single-site models were included in the MMM base model for each trait (Online Resource S6). In the base model, there were significant additive genetic variance components for GY at all sites except trial 2018AU2, and the average narrow-sense heritability for GY ranged widely across sites from 0.02 to 0.62 (mean 0.40) (Online Resource S6). Non-additive genetic variance for GY averaged 30.7% of total genetic variance and was similar in both Australia and Canada. Average narrow-sense heritability for other traits ranged from 0.44 (BL) to 0.83 (OL) (Online Resource S6).

Average accuracy ($r_{i}$) of additive genetic values across all individuals and all sites for GY in the base model was 0.826 in S_0_ genotypes and 0.843 in S_2+_ genotypes (an increase of 0.027). Average $r_{i}$ of additive genetic values in S_0_ genotypes exceeded 0.8 for all traits and exceeded 0.9 for DTF and OL (Online Resource S6).

#### 3.3.2. Optimum MMM-FA Models

For GY, the optimum MMM-FA model was FA(2), where the percentage genetic variance accounted for (%VAF) was 68.3% and there was a trivial increase in %VAF in the FA(3) model (Table 3). FA(2) was the optimum model for most traits except SW100 and DTF where the optimum model was FA(1) (Table 3). The average PBV for an individual across environments was generated from the optimum MMM-FA model for each trait at the end of cycle 4.

Most of the %VAF occurred in FA(1) with minor increases in FA(2), and for most traits, the loadings for FA(1) were all positive (Online Resource S8). In this case, OP is a “generalised main effect which allows for heterogeneity of scale between environments” [22], and OP is a valid measure of the overall genetic value of a variety for most traits. The exception is SW100, where there were negative and positive loadings across sites in FA(1) as a result of crossover genotype × environment interaction (Online Resource S8).

#### 3.3.3. Genetic Correlations across Trials 

**Figure 5:**
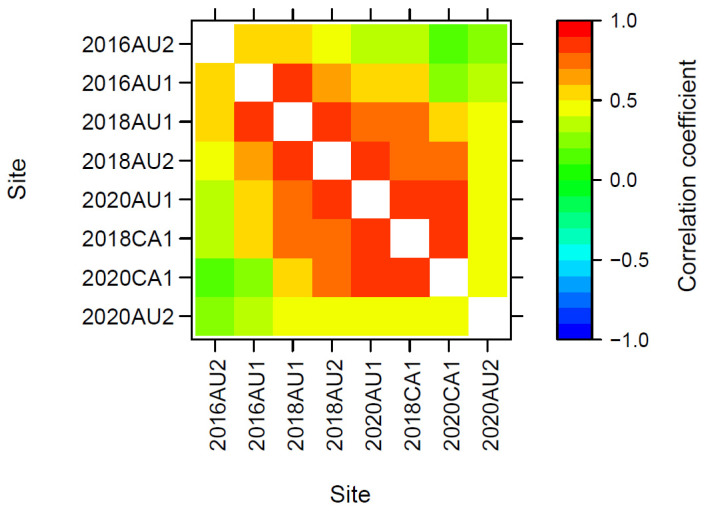
Genetic correlations of additive effects for grain yield across trial sites from the optimum MMM-FA model of the genotype × environment effects (see Table 2). For description of trial codes, see Table 1.

There were moderate to high genetic correlations of additive effects for GY across trials in Australia and Canada within and between cycles (Figure 5) and high correlation coefficients across trials for all other traits (Online Resource S9). That is, trials in Canada and Australia identified similar high-performing and low-performing genotypes for all traits.

The genetic correlations of GY between sites in Canada and Australia in cycle 2 was 0.80 (2018CA1 vs. 2018AU2) and 0.73 (2018CA1 vs. 2018AU1), and 0.81 between the two Australian sites in cycle 2 (2018AU1 vs. 2018AU2) (Figure 5, Online Resource S9). Likewise, the genetic correlations of GY across cycles 3 and 4 in Canada (2018CA1 vs. 2020CA1) was 0.84, between Canada and Australia was 0.84 (2018CA1 vs. 2020AU1) and within Australia was 0.79 (2018AU1 vs. 2020AU1) (Figure 5, Online Resource S9). 

Genetic correlations of GY between the disease nursery sites 2016AU2 and 2020AU2 and other sites in Australia or Canada were low to moderate, whereas genetic correlations of GY between non-disease sites were much higher (Figure 5, Online Resource S9). This may result from some BL-susceptible genotypes performing relatively poorly for GY at disease nursery sites but relatively better at non-disease nursery sites, and vice versa.

### 3.4. Pairwise Correlations of PBV across Traits 

**Figure 6:**
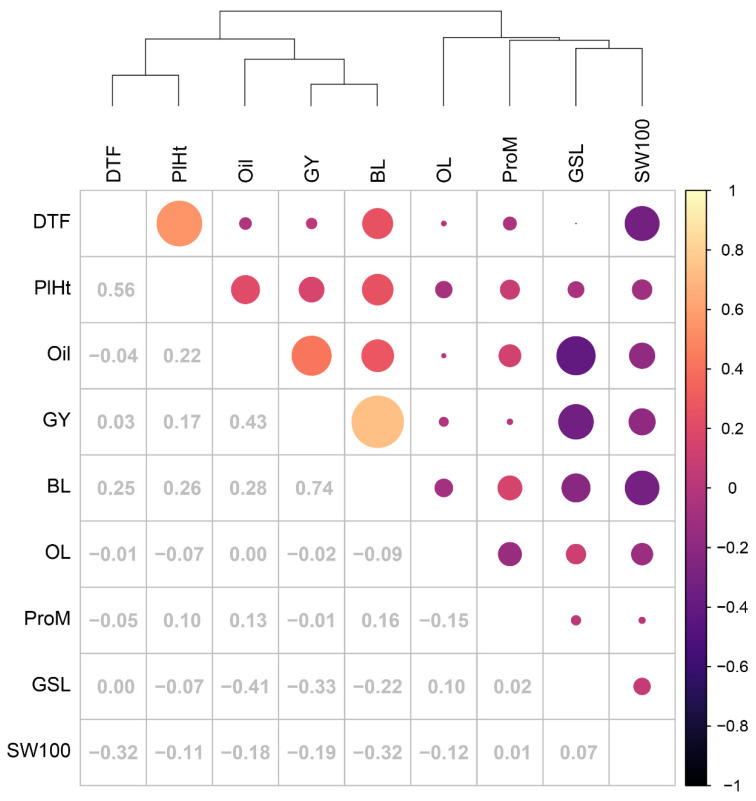
Cluster analysis of pair-wise correlations of predicted breeding values (PBV) derived from the optimum MMM-FA model of the genotype × environment effects for each trait. The traits correspond to grain yield (GY), days to 50% flower (DTF), plant height (PlHt), seed oil (Oil), protein in meal (ProM), glucosinolates (GSL), oleic acid (OL), phoma stem canker (blackleg) disease score (BL) and 100 seed weight (SW100). Correlation coefficients are shown as values below the diagonal and as circles above the diagonal, where the relative size and colour of circles represents the size and sign of the correlation coefficients as indicated by the colour code on the right-hand side.

Cluster analysis of pair-wise correlations of PBV across traits from the optimum MMM-FA model revealed two main groups of traits (Figure 6). GY, BL, Oil, PlHt, and DTF clustered together as a group of positively correlated traits, and this group tended to be negatively correlated with a second group of traits including GSL, ProM, OL, and SW100. DTF was strongly positively correlated with PlHt and late flowering lines tended to have smaller seeds (Figure 6). GY, Oil, PlHt, and DTF were all positively correlated with BL, that is, BL-resistant genotypes tended to be high-yielding, later flowering, and taller with higher seed oil. GSL was negatively correlated with both Oil and GY (Figure 6).

### 3.5. PBV, Overall Performance and Yield Stability for GY

**Figure 7:**
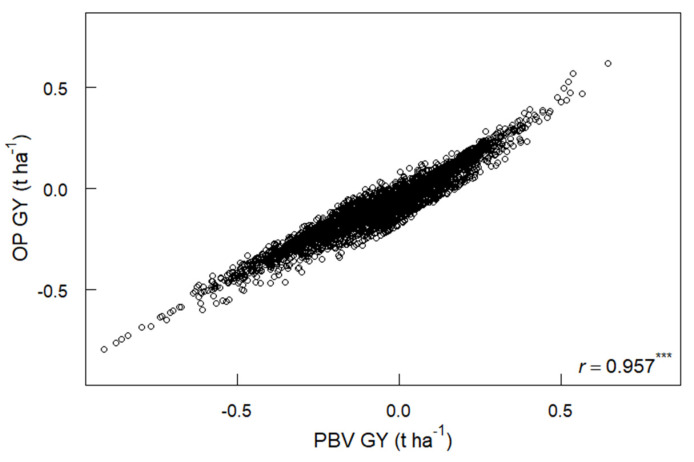
Correlation of overall performance for grain yield (OP GY, t ha^−1^) and predicted breeding values for GY (PBV GY, t ha^−1^) across genotypes from the optimum MMM-FA model (see Table 2). *** *p* < 0.001.

PBV GY and OP GY from the optimum MMM-FA model were closely correlated across genotypes (*r* = 0.957), which confirms that both assess average performance of genotypes although the range of values was wider for PBV GY than for OP GY (Figure 7).

RMSD values for GY (t ha^−1^) were perfectly correlated to the absolute value of *f*_2_ scores as expected from the FA(2) MMM-FA model for GY [21,22], although this may not necessarily be the case for FA(3) and higher-level models. RMSD is preferred over f_2_ scores since it is scaled in trait units and is applicable to FA models at all levels [22].

### 3.6. Genetic Gain across Cycles 

**Figure 8:**
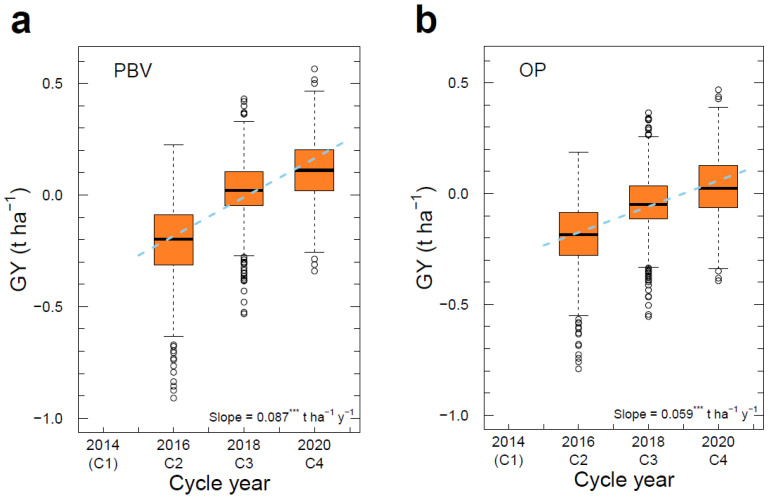
Genetic gain per year for grain yield (GY, t ha^−1^) based on the slope of the linear regression expressed in t ha^−1^ y^−1^ of (**a**) PBV averaged across environments of candidates in cycle 2 (2016 C2), cycle 3 (2018 C3) and cycle 4 (2020 C4), and (**b**) OP values over cycle 2, cycle 3 and cycle 4. Box plots represent the range of PBV and OP of candidates in each cycle. PBV and OP were obtained from the optimum MMM-FA model of the genotype × environment interaction effects. Data from cycle 1 (2014 C1) were excluded from the analysis, so C1 is shown in parentheses. Significance of the slope of the linear regressions: *** *p* < 0.001.

There was a very high rate of genetic gain in GY in the breeding population of 0.087 t ha^−1^ y^−1^ or 4.3% y^−1^ (expressed as a percentage of the population mean) over cycles 2, 3, and 4 from 2016 to 2020 as assessed by PBV for candidates for crossing in each cycle (Figure 8a, Table 4). Genetic gain assessed by OP resulted in a lower slope of 0.059 t ha^−1^ y^−1^ or 2.9% y^−1^ over cycles 2, 3, and 4 from 2016 to 2020, mostly due to lower cycle 3 and cycle 4 means in OP (Figure 8b) compared with PBV (Figure 8a).

The significant genetic gain in GY over cycles (Figure 8) occurred in the absence of any environmental trend in site mean GY across cycles (Figure 4). Therefore, the estimates of genetic gain in GY over cycles (Figure 8) were not influenced by environmental trends and are true genetic trends. 

There was a very high rate of genetic gain in the economic index of 7.26% y^−1^ (US$69.96 ha^−1^ y^−1^) as assessed by PBV and 5.05% y^−1^ (US$47.71 ha^−1^ y^−1^) as assessed by OP over cycles 2, 3 and 4 from 2016 to 2020 (Table 4, Online Resource S10). 

Significant genetic gain (or loss) in PBV and OP was found for all traits under selection in the economic index except PlHt (Table 4, Online Resource S10). There were no environmental trends in site means across cycles for any trait except for a very small environmental trend upwards in SW100 across cycles (Online Resource S7) which was opposed to the small genetic trend downwards in SW100 across cycles (Table 4). Therefore, the estimates of genetic trend in PBV over cycles for all traits are true genetic trends. 

The economic weight in the selection index for increasing ProM was double that for increasing Oil (Table 2) in order to achieve the desired positive gains in both ProM (+0.167% y^−1^) and Oil (0.276% y^−1^) when assessed by PBV. The genetic gain in ProM assessed by OP (+0.187% y^−1^) was superior to the assessment by PBV, but the genetic gain in Oil by OP (0.249% y^−1^) was inferior to the assessment by PBV (Table 4).

The average BL score in the population began at a low level in cycle 2 but increased rapidly by +8.3% y^−1^ as assessed by PBV, and +7.0% y^−1^ as assessed by OP (Table 4, Online Resource S10).

### 3.7. Genetic Gain in Historical Cultivars

**Figure 9:**
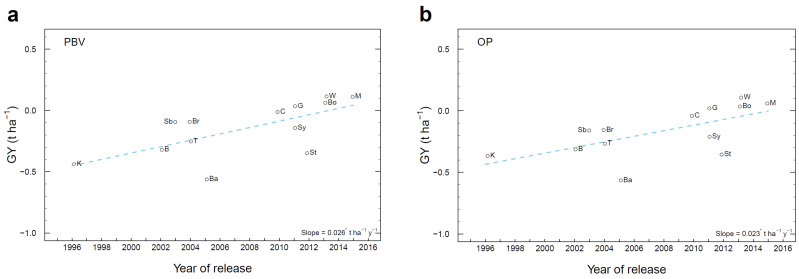
Genetic gain per year for grain yield (GY, t ha^−1^) in historical cultivars in the trials, based on the slope of the linear regression across year of release expressed in t ha^−1^ y^−1^ of (**a**) PBV for GY averaged across environments where the mean standard error of cultivars was 0.133 t ha^−1^, and (**b**) OP for GY. PBV and OP were obtained from the optimum MMM-FA model of the genotype × environment interaction effects. Significance of the slope of the linear regressions: * *p* < 0.05. Abbreviations of historical cultivars and year of release: K (Karoo, 1996), B (ATR Beacon, 2002), Sb (ATR Stubby, 2003), Br (Bravo TT, 2004), T (Tornado TT, 2004), Ba (Banjo TT, 2005), C (Crusher TT, 2010), G (ATR Gem, 2011), Sy (ATR Stingray, 2011), St (Sturt T, 2012), A (ATR Wahoo, 2013), Bo (ATR Bonito, 2013), and M (ATR Mako, 2015).

The rate of genetic gain in GY in historical cultivars was 0.026 t ha^−1^ y^−1^ (1.3% y^−1^) over their year of release from 1996 to 2015 as assessed by PBV (Figure 9a), and 0.023 t ha^−1^ y^−1^ (1.1% y^−1^) as assessed by OP (Figure 9b). 

Neither Oil nor ProM changed significantly over the year of release in historical cultivars, although the trend was towards higher Oil and lower ProM (Online Resource S10). There were no significant trends in BL, PlHt, and DTF over the year of release, but large variation between cultivars (Online Resource S10). 

In contrast, the breeding population showed significant genetic gains in PBV over cycles for most traits, although in the case of GY and BL, the breeding population began below the average of historical cultivars in cycle 2 and exceeded historical cultivars by cycle 4 (Figure 8 and Figure 9, Online Resource S10).

### 3.8. Impact of Genetic Correlations between Traits on Genetic Gain 

The strong positive genetic correlations between BL, GY, DTF and PlHt resulted in the population becoming taller and later flowering in cycles 2 and 3 (Figure 6, Online Resource S10). This group of traits was negatively correlated with ProM, and therefore the economic weight for ProM was double that for Oil (Table 2) in order to achieve desired genetic gains in ProM and Oil and all other traits. It was also necessary to introduce a negative economic weight against PlHt and DTF in cycles 3 and 4 (Table 2), and as a result, these began to fall in cycle 4 (Online Resource S10). The negative correlation between GY and GSL (Figure 6), coupled with the positive genetic gain in GY and Oil, contributed to a significant decrease in GSL over cycles (Online Resource S10). 

### 3.9. Predictions from OCS in Cycle 5 with and without RMSD GY in the Economic Index 

Two scenarios were assessed for the selection of parents and optimised mating design in MateSel at the end of cycle 4: an economic index excluding RMSD GY, and an economic index including a negative economic weight on RMSD GY (Table 5). The goal of including RMSD GY in the index was to improve yield stability (that is, to decrease RMSD GY) in the population and to evaluate the impact RMSD GY on the genetic gain in GY and other traits.

**Figure 10:**
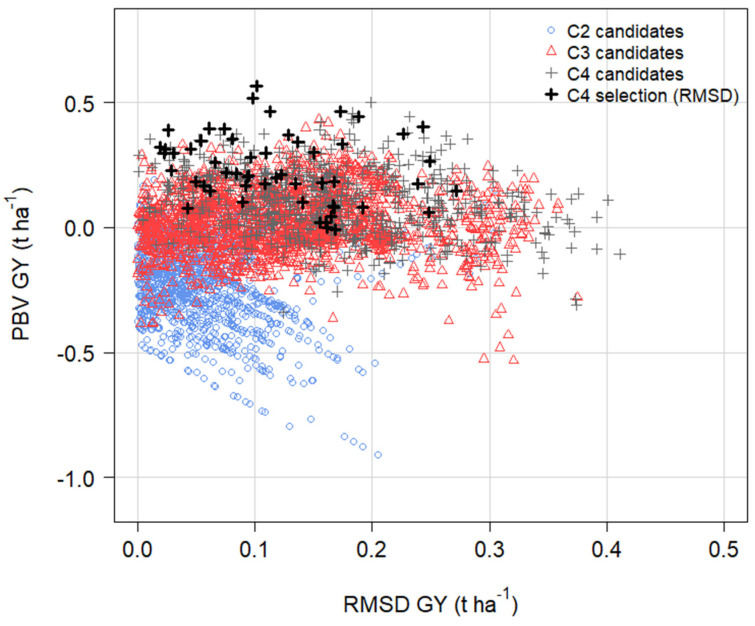
Change in predicted breeding values for grain yield (PBV GY, t ha^−1^) and root-mean-square deviation for grain yield (RMSD GY, t ha^−1^) of candidates for selection in cycle 2 (C2), cycle 3 (C3) and cycle 4 (C4) from the optimum MMM-FA model of the genotype × environment effects when RMSD GY was included in the economic index. The cycle 4 candidates selected as parents for crossing in OCS to begin cycle 5 are marked in bold black ‘+’ symbols.

In cycles 3 and 4, RMSD GY ranged widely from close to zero (stable across environments) to 0.40 t ha^−1^ (unstable across environments) with no signs of decreasing across cycles, while PBV GY in the population increased (Figure 10). With a negative economic weight on RMSD GY in the index and an optimum mating design generated in OCS, the population mean RMSD GY was predicted to decrease from 0.160 t ha^−1^ in cycle 4 to 0.109 t ha^−1^ in cycle 5, equivalent to 32.2% reduction or 16.1% y^−1^ (Table 5). As a result, the candidates selected for crossing in cycle 4 in MateSel tended to have lower RMSD GY (higher yield stability across environments) and higher PBV GY than non-selected candidates (Figure 10). 

The predicted increase in PBV GY from cycle 4 to cycle 5 was 0.164 t ha^−1^ or 4.1% y^−1^ relative to the population mean without RMSD GY in the index, and 0.170 t ha^−1^ or 4.2% y^−1^ with RMSD GY in the index (Table 5). That is, the inclusion of RMSD GY in the index had no negative impact on the genetic gain in GY or other traits except for DTF and PlHt, which had a slower response to selection for earlier and shorter types when RMSD GY was included in the index (Table 5). Interestingly, even without RMSD GY in the index, RMSD GY was predicted to decrease by 19.8% in cycle 5, which suggests that high GY is associated with a more stable GY when selection is based on OCS with optimum mating designs (Table 5).

There was a small increase in achieved parental coancestry in cycle 5 from 0.085 without RMSD GY to 0.087 when RMSD GY was included in the index (Table 5). This is a very low level of achieved parental coancestry in cycle 5. We conclude that the inclusion of RMSD GY in the index did not limit potential genetic gain for GY and other key traits in cycle 5.

The population mean PBV of both DTF and PlHt was predicted to continue to decline in cycle 5 (Table 5) in response to negative economic weights in the index (Table 2). Mean PBV BL in the population was predicted to increase only marginally in cycle 5 (Table 5, Online Resource S10).

## 4. Discussion

A very high rate of genetic gain in GY of 0.087 t ha^−1^ y^−1^ (4.3% y^−1^) was achieved inside a spring oilseed rape (canola) breeding program based on field testing in Australia and Canada over three cycles of S_0,1_ family selection from 2016 to 2020, as assessed by average predicted breeding values (PBV) for GY in the optimum MMM-FA model (Figure 8a). The rate of gain assessed by overall performance (OP) based on f_1_ scores in the optimum MMM-FA model and expressed in trait units was lower at 0.059 t ha^−1^ y^−1^ (2.9% y^−1^) (Figure 8b). This very high rate of genetic gain in GY in spring canola was the result of index selection of multiple economic traits with optimized mating designs based on OCS, and was predicted to continue with low rates of achieved parental coancestry in cycle 5 (Table 5). This high rate of genetic gain has the potential to continue into the foreseeable future as anticipated in stochastic models based on this breeding approach [9,10].

Both measures of genetic gain, PBV and OP, are valid and interesting outcomes of MMM-FA models, which help to explain the environmental impact on the assessment of genetic gain. Both PBV and OP support the conclusion that rapid genetic gain in spring oilseed rape was achieved across two disparate global regions (summer growing season in Canada; winter-spring growing season in Australia). All the evidence, including high genetic correlations of additive effects across trials for most traits, suggests that the genetic gains in this population were realised in both Australia and Canada.

The reasons for the lower rate of genetic gain assessed by OP vs. PBV in this study are complex but may reflect the relatively low loadings to FA(1) for GY in the optimum MMM-FA model allocated to disease nursery trials 2018AU2 and 2020AU2 in cycles 3 and 4 (Online Resource S8). At these disease nursery sites, BL-resistant candidates with relatively high GY may contribute more to PBV than to OP as a result of the low loadings to FA(1) for GY at these sites. Consequently, PBV would be higher than OP for the candidates in cycles 3 and 4 (Figure 8). 

The genetic gain was achieved in several agronomic and grain quality traits in these trials, including increases in Oil, ProM, and BL disease resistance and a decrease in GSL (Table 4). These estimates of genetic gain were not biased by environmental trends over years [19,20].

In historical cultivars in the same trials, the estimate of genetic gain over years of release was considerably less than inside the breeding population. Genetic gain in GY in Australian historical triazine tolerant cultivars was 0.026 t ha^−1^ y^−1^ (1.3% y^−1^) from 1996 to 2015 as assessed by PBV (Figure 9a), and 0.023 t ha^−1^ y^−1^ (1.1% y^−1^) as assessed by OP (Figure 9b). These values are close to the genetic gain in historical cultivars of lupins, which were 0.020 t ha^−1^ y^−1^ over 31 years assessed by PBV and 0.017 t ha^−1^ y^−1^ assessed by OP [21]. Similarly, the average achieved a genetic gain in GY in the world’s major self-pollinating grain crops is approximately 1% y^−1^ [1]. 

High rates of genetic gain for GY and other traits inside this actively evolving crop breeding program in Australia and Canada verify the predictions of stochastic models of augmented S_0,1_ family selection with OCS based on an economic index [9,10]. Also, for the first time in crop breeding, we incorporate a parameter related to yield stability (RMSD) [22] in the economic index in cycle 4, and RMSD GY was predicted to decrease (that is, GY stability to increase) in cycle 5 without negatively impacting the rate of genetic gain in GY (Table 5). This result, and the high genetic correlations (>0.80) in GY and all other traits across sites in Canada and Australia (Figure 5, Online Resource S9), support the conclusion that yield stability across both local and global environments can be improved with the inclusion of RMSD in the economic index.

In addition to high rates of genetic gain in GY, rapid increases were achieved in Phoma stem canker (blackleg) disease resistance (+8.3% y^−1^), seed oil (+0.62% y^−1^), and protein in meals (+0.41% y^−1^), while seed glucosinolate content decreased (−3.4% y^−1^) (Table 4). However, genetic correlations between traits were both helpful and detrimental, and changes were made to the weightings of traits in the economic index across cycles as more knowledge became available to achieve desired gains. OCS in MateSel was important to evaluate the best weighting on traits in the index to achieve the desired predicted genetic gains in the next cycle, for example, to increase the weighting against tall and late flowering genotypes in the index for the selection of parents for cycle 5 (Table 2). This resulted in a predicted decrease in the population mean DTF and PlHt in cycle 5 (Table 5, Online Resource S10).

These high rates of genetic gain in GY and other traits in the economic index occurred with an achieved parental coancestry in cycle 5 parents of 0.087 with RMSD GY in the index (Table 5). This was lower than predicted in stochastic models of augmented S_0,1_ family selection with OCS based on an economic index, where achieved parental coancestry was >0.20 with OCS after five cycles [10]. In this study, 35 new migrants (F_1_’s of SH × NH crosses) were added in cycles 1, 2, 3, and 4, which helped to reduce achieved parental coancestry in cycle 5 (Online Resource S2). The low achieved parental coancestry in cycle 5 bodes well for future genetic gain in this breeding program, which should continue at similarly high rates for 20 or 30 cycles as predicted in stochastic models [9,10].

The breeding method outlined here follows the principles of BRIO crop breeding, based on accurate breeding values, rapid cycles, index selection, and OCS [52], and adherence to these principles was important to achieve rapid and sustainable improvements in several economic traits in this study. OCS or similar optimised mating systems are important to conserve genetic diversity while optimising long-term genetic gain [53,54]. BRIO principles are flexible to include new technology as it arises. For example, we applied these principles to the breeding of a common bean with genomic relationship information [32]. Genomic relationship information (G-BLUP) may be combined with additive relationship information (A-BLUP) in single-step H-BLUP analysis [31] which should further improve the accuracy of the PBV and help to accelerate genetic gain. Another attractive feature of BRIO principles is that selection goals can be adapted to changing circumstances over time, just as we adapted selection goals to react to undesirable PlHt and DTF in cycles 2 and 3. In the future, we may include heat stress tolerance in the index to pre-empt the negative impact of global warming on GY [10].

The size and cost of this augmented S_0,1_ family selection breeding program were relatively small—a total of 900 crosses were undertaken and 14,424 small field plots were sown and harvested in field plot trials at 9 locations in Australia and Canada from 2014 to 2020 (Table 2). The cost of the field plots and grain quality testing is the same as in traditional plant breeding trials. The analyses we applied to augmented S_0,1_ family selection, including MMM-FA analysis and OCS in MateSel, were carried out on a laptop computer. OCS in MateSel was used to improve GY, cooking time, and seed Fe and Zn content in a common bean breeding program in East Africa [32] and to improve different traits in Desi and Kabuli chickpea varieties [55]. Another positive feature of augmented S_0,1_ family selection is that it delivers inbred lines for advanced testing as shown in the pedigree diagram (Online Resource S1) and thereby combines the population improvement phase with the product development phase of plant breeding [56]. 

Interestingly, we achieved only a small increase in the accuracy of additive genetic values in inbred lines over non-inbred genotypes of approximately 0.02 (Online Resource S6), which supports the use of non-inbred parents to reduce breeding cycle time [5]. Our results show that selfing to purity is not essential before selection and use as a parent and may slow the rate of genetic gain by extending the generation interval (L). One reason for the relatively high accuracy of additive genetic values in non-inbred lines in this study is that we carried forward both self and cross progeny into the next cycle, and this increased the number of collateral relatives within cycles and increased the connectivity of genetic relationships across cycles. Cross-and-self-sibs have a coefficient of coancestry (*f*) equivalent to full-sibs which helps to increase the accuracy of PBV.

MMM-FA provides a means to assess yield stability (RMSD GY) and overall performance (OP) of genotypes in the presence of genotype by environment interactions [21,22]. OP is a useful concept when a diverse breeding population is evaluated over multiple sites and cycles, and genotype by environment interactions are relatively low compared to the generalised main effect of variety performance [22]. PBV GY values of genotypes in the optimised MMM-FA model were closely correlated to OP GY values in this study (*r* = 0.957) (Figure 7), and both PBV and OP are valid measures of genetic gain. RMSD GY in this study reflects global yield stability across multiple sites in Australia and Canada. RMSD GY was perfectly associated with f_2_ scores for GY in the FA(2) model in this study but this may not be the case in higher-order FA models.

Future selection for adaption to global spring rapeseed environments may require economic weights on traits specific to regions (for example, there may be specific weights for important spring canola target regions such as Canada, Europe, China, India, and/or Australia), and this may be accommodated through ‘multiple end-uses’ in MateSel [26]. The population would be managed with multiple versions of the economic selection index for each target region and a global index applicable to all regions, with specific crossing programs targeted for each region.

The integration of multiple traits into an economic index follows the logic of Hazel and Lush [57] that selection on an “index of net desirability is much more efficient than selection for one trait at a time”. However, index selection must be accompanied by OCS or a similar optimised mating system to optimize long-term genetic gain. OCS based on an economic index was essential in this study to achieve concurrent genetic improvements in GY, Oil, ProM, and BL while decreasing GSL and maintaining OL, SW100, DTF, and PlHt with low rates of population inbreeding. The breeder can change economic weightings in the economic index to achieve desired gains over cycles as more data become available or breeding goals change; for example, here we increased the index weightings against DTF and PlHt in cycle 4 (Table 3) which greatly reduced the predicted population mean of these two traits in cycle 5 (Table 5). We also increased the index weighting of ProM to double the economic value of Oil (Table 3) in order to achieve a genetic gain in both traits (Online Resource S10). This is clearly a subjective decision of the breeder since there are no current economic incentives to improve ProM in canola, but this may change in the future [58] and appropriate weightings in the index selection together with OCS should achieve a long-term genetic gain in both attributes. Likewise, the economic weight on OL was zero in this study (Table 2), but it may be important to introduce a small economic weight to prevent OL from decreasing in the future in this population (Online Resource S10).

Rapid cycles of S_0,1_ family selection with OCS resulted in very high rates of genetic gain in GY and other commercial traits in spring oilseed rape in both Australia and Canada, thereby confirming the predictions of stochastic modelling of the same process with OCS and optimised mating designs [9,10]. Yield stability across these global environments was predicted to improve rapidly when RMSD GY was included in the economic index. OCS will help to accelerate genetic gain inside crop breeding programs while conserving genetic diversity to assure future genetic gains [54], and thereby help to meet the expected global demand for grains as food and feed in 2050 [1,2,3].

## Figures and Tables

**Table 1:** (**a**) Cycle number, trial code, trial location and country of each trial site, the number of plots, ranges, rows, genotypes (including number tested in a single replicate) in each trial, and (**b**) connectivity of genotypes across trials in the study. Genotypes defined as S_0_ were tested in field trials as S_0,1_ bulks; likewise F_2_ genotypes were tested as F_2,3_ bulks, S_2_ genotypes were tested as S_2,3_ bulks, and so on. State abbreviations (Australia): Western Australia (WA), Victoria (VIC). Province abbreviation (Canada): Manitoba (MB).

(a)														
							Number of Genotypes in Trial							
Cycle	Trial Code	Trial Location	Country	Ranges	Rows	Plots	Total	Single Replicate	S_0_	S_1_	S_2_	S_4_	F_2_	F_3_
1	2014AU1	West Dale, WA	Australia	24	89	2136	1557	1080	668	250	0	0	577	52
2	2016AU1	York, WA	Australia	24	68	1632	1175	898	511	0	569	0	11	55
2	2016AU2	Rutherglen, VIC	Australia	24	68	1632	1174	899	511	0	568	0	11	55
3	2018AU1	York, WA	Australia	24	88	2112	1919	1769	1565	0	151	166	13	0
3	2018AU2	Rutherglen, VIC	Australia	24	88	2112	1912	1771	1566	0	151	166	13	0
3	2018CA1	Sun Valley, MB	Canada	24	88	2112	1919	1772	1566	0	151	166	13	0
4	2020AU1	Williams, WA	Australia	24	40	960	674	436	653	0	0	0	0	0
4	2020AU2	Wonwondah, VIC	Australia	24	38	912	625	386	604	0	0	0	0	0
4	2020CA1	Sun Valley, MB	Canada	24	34	816	555	308	528	0	0	0	0	0
**(b)**														
			**Connectivity of Genotypes across Trials**											
**Cycle**	**Trial Code**		**2014AU1**	**2016AU1**	**2016AU2**	**2018AU1**	**2018AU2**	**2018CA1**	**2020AU1**		**2020AU2**		**2020CA1**	
1	2014AU1		1556	3	3	2	2	2	1		1		1	
2	2016AU1		3	1175	886	13	13	13	5		5		5	
2	2016AU2		3	886	1174	13	13	13	5		5		5	
3	2018AU1		2	13	13	1911	1911	1911	4		4		4	
3	2018AU2		2	13	13	1911	1912	1912	4		4		4	
3	2018CA1		2	13	13	1911	1912	1919	4		4		9	
4	2020AU1		1	5	5	4	4	4	674		625		548	
4	2020AU2		1	5	5	4	4	4	625		625		548	
4	2020CA1		1	5	5	4	4	9	548		548		555	

**Table 2:** Calculation of economic index based on economic weights for traits to achieve desired genetic gains in a hypothetical genotype in cycle 4. The assumed grain price is US$550 t^−1^. The economic weight of a trait for +1 unit predicted breeding value (PBV) is shown as % grain price and in US$ t^−1^. The contribution of a trait to the economic index in US$ ha^−1^ is calculated by multiplying the economic weight for +1 PBV unit (US$ t^−1^) × PBV of the individual in units of trait × total GY of individual (t ha^−1^).

Trait ^a^	Population Mean (Units of Trait)	Range of PBV in Population (Units of Trait)		PBV of the Individual (Units of Trait)	Total GY of Individual (t ha^−1^)	Grain Price (US$ t^−1^)	Economic Weight for +1 PBV Unit		Contribution to Economic Index of the Individual (US$ ha^−1^)	Calculation of Economic Index (US$ ha^−1^) (Based on Economic Weight for +1 PBV in US$ t^−1^)
		Min	Max				% Grain Price	US$ t^−1^		
GY	2.020	−0.910	+0.644	+0.322	2.342	550.00			$1288.10	(Total GY of Individual) × (Grain Price)
Oil	44.757	−6.673	+3.686	+1.383			1.50%	$8.25	$26.72	(PBV Oil) × (Total GY) × (Econ Wt +1 unit PBV)
ProM	41.088	−4.062	+5.508	+0.554			3.00%	$16.50	$21.41	(PBV ProM) × (Total GY) × (Econ Wt +1 unit PBV)
DTF	80.180	−13.507	+15.639	+2.213			−1.00%	−$5.50	−$28.51	(PBV DTF) × (Total GY) × (Econ Wt +1 unit PBV)
PlHt	122.675	−29.380	+26.952	+3.561			−0.50%	−$2.75	−$22.93	(PBV PlHt) × (Total GY) × (Econ Wt +1 unit PBV)
BL	5.089	−1.996	+2.671	+1.297			2.00%	$11.00	$33.41	(PBV BL) × (Total GY) × (Econ Wt +1 unit PBV)
GSL	11.297	−5.637	+16.325	−1.749			−1.50%	−$8.25	$33.79	(PBV GSL) × (Total GY) × (Econ Wt +1 unit PBV)
SW100	0.325	−0.030	+0.039	+0.024			0.20%	$1.10	$0.06	(PBV SW100) × (Total GY) × (Econ Wt +1 unit PBV)
OL	61.670	−8.132	+8.939	+2.328			0.00%	$0.00	$0.00	(PBV OL) × (Total GY) × (Econ Wt +1 unit PBV)
RMSD GY	0.096	0.000	+0.411	+0.215			−20.0%	−$110.00	−$55.39	(PBV RMSD GY) × (Total GY) × (Econ Wt +1 unit PBV)
Economic index excluding RSMD GY									$1352.06	sum of above excluding RMSD GY (t ha^−1^)
Economic index including RMSD GY									$1296.67	sum of above including RMSD GY (t ha^−1^)

^a^ Trait abbreviations and units: GY = grain yield (t ha^−1^), DTF = days to 50% flowering, PlHt = plant height (cm), Oil = seed oil (%) at 6% moisture, ProM = protein in meal (%) at 10% moisture, GSL = glucosinolates (μmole g^−1^ seed), OL = oleic acid (%), BL = Phoma (blackleg) disease score from 1 (very susceptible) to 9 (very resistant), SW100 = 100 seed weight (g), and RMSD GY = root mean standard deviation for GY (t ha^−1^).

**Table 3:** Evaluation of MMM-FA models of the genotype × environment interaction effects for each trait, and number of estimated variance components in each model. The optimum model ^a^ was selected on the basis that it increased percentage genetic variance accounted for (%VAF), and significantly increased REML log-likelihood (RLL) and decreased Akaike information criterion (AIC) and Bayesian information criterion (BIC). The base model included information from the additive genetic relationship matrix and significant fixed and random terms.

MMM-FA Model	Number of Estimated Variance Components	RLL	AIC	BIC	%VAF
Grain yield (t ha^−1^)					
Base	44	8455.622	−16,823.2	−16,497.2	
FA(1)	53	8635.382	−17,164.8	−16,772.1	63.31
FA(2) ^a^	59	8647.610	−17,177.2	−16,740.1	68.33
FA(3)	65	8656.074	−17,182.2	−16,700.5	70.97
Days to 50% flower					
Base	25	−11,863.700	23,777.41	23,950.84	
FA(1) ^a^	31	−11,642.660	23,347.32	23,562.38	96.22
FA(2)	34	−11,638.730	23,345.45	23,581.33	96.40
Plant height (cm)					
Base	24	−15,452.09	30,952.18	31,110.93	
FA(1)	29	−15,392.17	30,842.34	31,034.16	31.83
FA(2) ^a^	31	−15,382.06	30,826.13	31,031.18	93.52
Seed oil (%) at 6% moisture					
Base	34	−8169.558	16,407.12	16,649.38	
FA(1)	41	−7836.272	15,754.54	16,046.69	81.46
FA(2) ^a^	45	−7825.844	15,741.69	16,062.33	94.57
Protein in meal (%) at 10% moisture					
Base	33	−7462.394	14,990.79	15,225.92	
FA(1)	40	−7189.898	14,459.80	14,744.80	32.24
FA(2) ^a^	44	−7188.877	14,465.75	14,779.26	84.99
Glucosinolates (μmole g^−1^ seed)					
Base	33	−12,630.21	25,326.41	25,561.54	
FA(1)	40	−12,121.96	24,323.92	24,608.93	75.34
FA(2) ^a^	44	−12,118.56	24,325.12	24,638.63	94.77
Oleic acid (% of total fatty acids)					
Base	22	−5873.146	11,790.29	11,938.68	
FA(1)	27	−5556.775	11,167.55	11,349.66	90.85
FA(2) ^a^	29	−5554.522	11,167.04	11,362.64	97.22
Phoma stem canker (blackleg) disease score: 1 (very susceptible) to 9 (very resistant)					
Base	16	−3164.365	6360.730	6463.844	
FA(1)	20	−3149.209	6338.417	6467.310	74.05
FA(2) ^a^	21	−3149.208	6340.417	6475.754	83.74
100 seed weight (g)					
Base	22	19,743.45	−39,442.91	−39,294.51	
FA(1) ^a^	27	19,808.11	−39,562.21	−39,380.09	51.93

^a^ Optimum MMM-FA model chosen for assessment of overall performance and predicted breeding values of genotypes.

**Table 4:** Genetic gain in predicted breeding values (PBV) and overall performance (OP) across cycles 2, 3 and 4 for each trait and the economic index. PBV and OP were obtained from the optimum MMM-FA model of the genotype×environment interaction effects (see Table 2). The linear regression coefficient of candidate PBV and OP across cycles 2 to 4 is shown in units cycle^−1^, and annual genetic gain from cycles 2 to 4 as units y^−1^ and as % of the population mean y^−1^ for each trait. Italicized values of coefficient and intercept in grey were not significant at *p* = 0.05.

Trait ^a^	Population Mean	Units	Method of Breeding Value Assessment	Mean PBV of Candidate Genotypes in each Cycle (Number of Candidates in each Cycle)			Linear Regression of PBV of Candidate Genotypes from Cycles 2 to 4		Annual Genetic Gain from Cycles 2 to 4^b^	
				Cycle 2 (1426)	Cycle 3 (1896)	Cycle 4 (653)	Coefficient	Intercept		
							Units Cycle^−1^ ± SE	Units ± SE	Change in Coefficient (units y^−1^)	Change in Coefficient (% y^−1^)
Index	963.8	US$ ha^−1^	PBV	857.309	994.828	1106.341	139.910 ± 2.424	593.210 ± 7.008	69.955	7.26
	944.6		OP	868.862	961.426	1061.069	95.430 ± 2.316	676.870 ± 6.696	47.714	5.05
GY	2.02	t ha^−1^	PBV	−0.207	0.026	0.113	0.1741 ± 0.0095	−0.5321 ± 0.0033	0.0870	4.31
			OP	−0.188	−0.04	0.032	0.1172 ± 0.0030	−0.4099 ± 0.0087	0.0585	2.90
DTF	80.2	days	PBV	1.872	2.135	0.474	−0.5150 ± 0.0932	3.2127 ± 0.2695	−0.258	−0.32
			OP	1.793	1.945	0.343	−0.5580 ± 0.0888	3.1916 ± 0.2568	−0.279	−0.35
PlHt	122.7	cm	PBV	0.254	2.923	−1.275	*−0.1711 ± 0.1776*	1.7305 ± 0.5133	−0.0860	−0.07
			OP	0.212	2.927	−1.419	*−0.1406 * *± * *0.1857*	1.6337 ± 0.5369	−0.0705	−0.06
Oil	44.8	%	PBV	−0.996	−0.366	0.070	0.5517 ± 0.0267	−2.0681 ± 0.0772	0.2760	0.62
			OP	−0.978	−0.421	−0.012	0.4970 ± 0.0261	−1.9479 ± 0.0754	0.2485	0.55
ProM	41.1	%	PBV	0.255	0.433	0.995	0.3331 ± 0.0268	−0.4730 ± 0.0776	0.167	0.41
			OP	0.256	0.508	1.059	0.3729 ± 0.0264	−0.5380 ± 0.0762	0.187	0.45
GSL	11.3	μmole g^−1^	PBV	0.314	−0.859	−1.030	−0.7680 ± 0.0452	1.6885 ± 0.1306	−0.384	−3.40
			OP	0.290	−0.910	−1.075	−0.7820 ± 0.0444	1.6868 ± 0.1284	−0.391	−3.46
OL	61.7	%	PBV	−0.194	−0.541	−0.645	−0.2483 ± 0.0433	0.2629 ± 0.1253	−0.124	−0.20
			OP	−0.181	−0.585	−0.671	−0.2755 ± 0.0431	0.3190 ± 0.1245	−0.138	−0.22
BL	5.1	units	PBV	−0.283	0.911	1.234	0.8417 ± 0.0151	−1.8255 ± 0.0437	0.421	8.25
			OP	−0.239	0.739	1.057	0.7114 ± 0.0136	−1.5556 ± 0.0393	0.356	6.97
SW100	0.325	g	PBV	−0.003	−0.005	−0.006	−0.0019 ± 0.0002	*0.0010 ± 0.0005*	−0.001	−0.30
			OP	−0.001	0.001	0.001	0.0008 ± 0.0000	−0.0020 ± 0.0001	0.001	0.15

^a^ Trait abbreviations: GY = grain yield, DTF = days to 50% flowering, PlHt = plant height, Oil = seed oil at 6% moisture, ProM = protein in meal at 10% moisture, GSL = glucosinolates, OL = oleic acid, BL = Phoma (blackleg) disease score from 1 (very susceptible) to 9 (very resistant), and SW100 = 100 seed weight. ^b^ Genetic gain in PBV and OP per year is calculated by dividing the linear regression coefficient across cycles by 2, and is expressed as units y^−1^ and as % of population mean y^−1^.

**Table 5:** (**a**) Output from MateSel with two optimal contribution selection (OCS) scenarios for crossing among cycle 4 parents: the economic index either includes or excludes yield stability, based on the root mean square deviation (RMSD) from the regression line associated with the first factor in the optimum MMM-FA model for grain yield (GY). The MateSel output is further explained in Kinghorn and Kinghorn (31). (**b**) Predicted response in cycle 5 with and without RMSD GY in the index.

(a) MateSel Output							
			Without RMSD GY in index	With RMSD GY in Index	Notes		
No. male candidates			653	653			
No. female candidates			653	653			
No. selected males			44	41	moderate weighting against reciprocal matings, selfings and duplicates		
No. selected females			37	39	moderate weighting against reciprocal matings, selfings and duplicates		
No. matings used			150	150			
Target degrees			45	45	conservative strategy to minimise coancestry and maximise index at 45 degrees		
Achieved degrees			45	45			
Achieved parental coancestry			0.0850	0.0867	low achieved parental coancestry		
Starting mean candidate index			1140.80	1106.34			
Achieved mean progeny index			1307.22	1282.83	aim to increase mean progeny index		
Achieved standard deviation progeny index			24.31	26.78			
Lowest selected male index			1135.55	1113.01			
Lowest selected female index			1121.90	1036.26			
Weighting on progeny mean inbreeding (*F*)			−1	−1			
Achieved progeny mean inbreeding (*F*)			0.0093	0.0103	low due to optimised mating scheme		
Random mating inbreeding (*F*)			0.0779	0.0803	predicted *F* based on random mating among parents		
Maximum inbreeding (*F*) achieved			0.0788	0.0843	achieved following optimised design		
**(b) Predicted Responses in cycle 5**							
			**Without RMSD GY in Index**		**With RMSD GY in Index**		
	**Units**	**Population Mean across Cycles 2, 3, 4**	**Predicted Response in** **Cycle 5 (Units)**	**Predicted Response in** **Cycle 5 as % Population Mean**	**Predicted Response in Cycle 5 (Units)**	**Predicted Response in** **Cycle 5 as % Population Mean**	**Selection Aim**
Economic index	US$ ha^−1^		166.42		176.49		increase
GY	t ha^−1^	2.017	0.1637	8.12%	0.1696	8.41%	increase
DTF	days	80.2	−2.5512	−3.18%	−2.1776	−2.72%	decrease
PlHt	cm	122.7	−2.9254	−2.38%	−2.6353	−2.15%	decrease
Oil	%	44.8	0.2626	0.59%	0.2816	0.63%	increase
ProM	%	41.1	0.4137	1.01%	0.4426	1.08%	increase
GSL	μmole g^−1^	11.3	−0.6780	−6.00%	−0.7987	−7.07%	decrease
OL	%	61.7	−0.0923	−0.15%	−0.2025	−0.33%	change
BL	scale 1−9	5.1	0.1308	2.57%	0.1820	3.57%	increase
SW100	g	0.325	0.0022	0.69%	0.0020	0.60%	increase
RMSD GY	t ha^−1^	0.160	−0.0317	−19.78%	−0.0514	−32.15%	decrease

## Data Availability

The data presented in this study are available on request from the corresponding author.

## Supplementary Materials

The following supporting information can be downloaded at: https://www.mdpi.com/article/10.3390/plants12020383/s1. Online Resource S1. Pedigree graph of a portion of the ancestral pedigree; Online Resource S2. Flow of germplasm during four two-year cycles of augmented S0,1 family selection; Online Resource S3. Inbreeding coefficient (*F*) of genotypes; Online Resource S4. Coefficient of coancestry (*f*) of related genotypes; Online Resource S5. Statistical models description; Online Resource S6. Summary of trial data for all traits; Online Resource S7. Environmental trends in site predicted means for traits; Online Resource S8. Loadings for FA(1) and FA(2) from the optimum MMM-FA model; Online Resource S9. Correlation of additive genetic effects across trials; Online Resource S10. Genetic changes in predicted breeding values (PBV) for all traits.
