# Supplementary material for: Optimal Contribution Selection Improves the Rate of Genetic Gain in Grain Yield and Yield Stability in Spring Canola in Australia and Canada
Source: Plants (Basel). 2023 Jan 13;12(2):383. doi: 10.3390/plants12020383 (PMC9863350; doi:10.3390/plants12020383)
Supplement: Supplementary file 1 [file plants-12-00383-s001.zip › plants-2061854-supplemental files/Online Resource S1 Pedigree diagram_Cowling et al_submit.pdf]

Rapid and stable global genetic gain in crop breeding: MDPI Plants

Wallace A Cowling*, Felipe Castro-Urrea, Katia T Stefanova, Li Li, Robert G Banks, Renu Saradadevi, Olaf Sass, Brian P Kinghorn & Kadambot H M Siddique
*Corresponding author, The University of Western Australia, wallace.cowling@uwa.edu.au

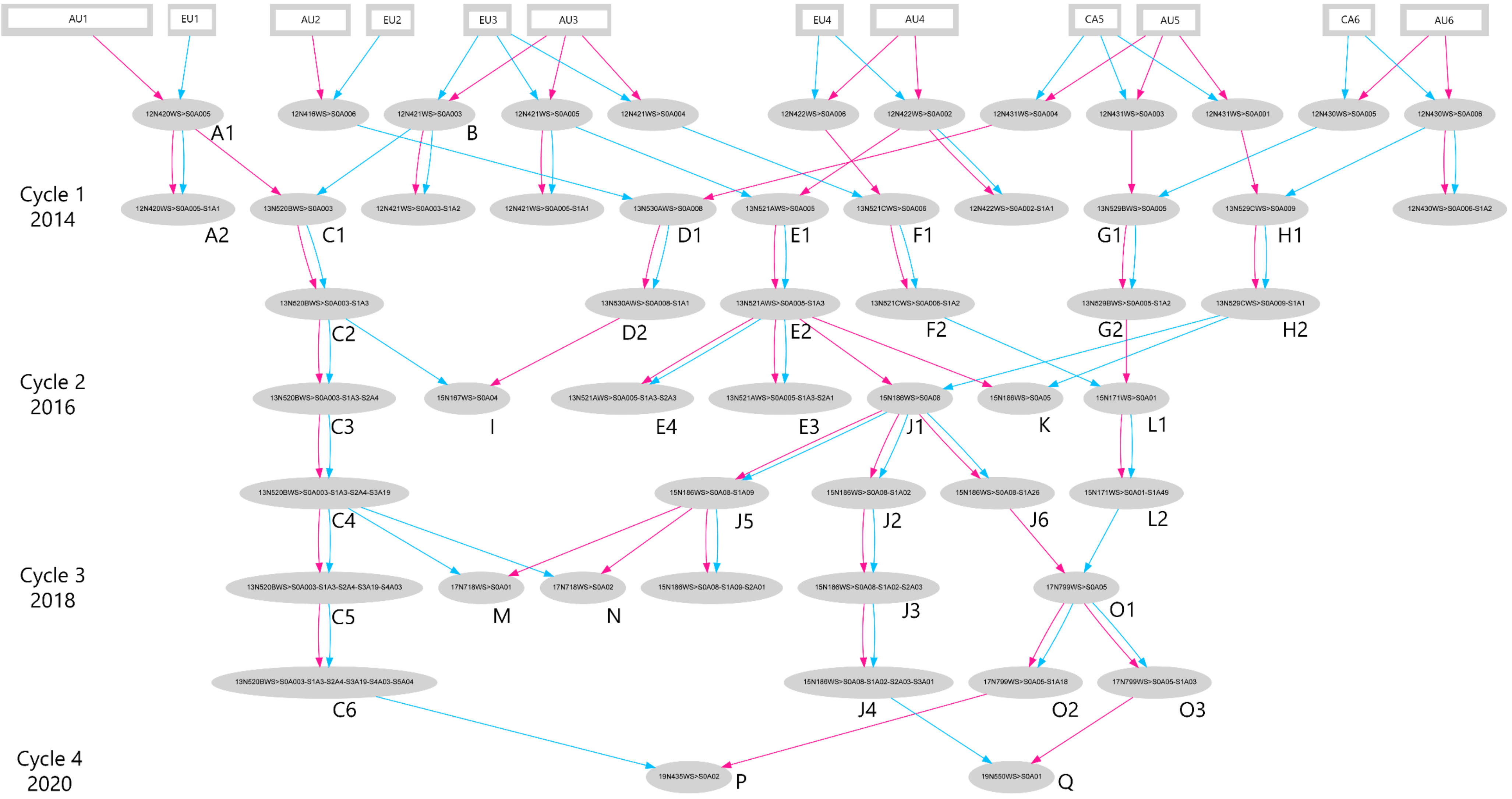

Online Resource S1. Pedigree graph of a portion of the ancestral pedigree arising from 12 founder lines from the northern hemisphere in Canada (CA) or Europe (EU) or from the southern hemisphere in Australia (AU). Founder lines were intercrossed CA×AU and EU×AU and their F_1 progeny were intercrossed to begin field testing of $S_{0,1}$ families in cycle 1 in 2014. Red arrows represent gametes from female parents, and blue lines are gametes from male parents. Selfing is indicated by both red and blue arrows linking parent and progeny genotypes. Progeny with code names are referred to in the text and in Online Resources 3 and 4.
