# Supplementary material for: Optimal Contribution Selection Improves the Rate of Genetic Gain in Grain Yield and Yield Stability in Spring Canola in Australia and Canada
Source: Plants (Basel). 2023 Jan 13;12(2):383. doi: 10.3390/plants12020383 (PMC9863350; doi:10.3390/plants12020383)

Rapid and stable global genetic gain in crop breeding - MDPI Plants

Wallace A Cowling*, Felipe Castro-Urrea, Katia T Stefanova, Li Li, Robert G Banks, Renu Saradadevi, Olaf Sass, Brian P Kinghorn & Kadambot H M Siddique
*Corresponding author, The University of Western Australia, wallace.cowling@uwa.edu.au

Online Resource S10. Genetic changes in predicted breeding values (PBV) for (**i**) economic index (US$ ha^{-1}), (**ii**) days to 50% flower (DTF, days), (**iii**) plant height (PlHt, cm), (**iv**) seed oil (Oil, %) at 6% moisture, (**v**) protein in meal (ProM, %) at 10% moisture, (**vi**) glucosinolates in seed (GSL, µmole g^{-1}), (**vii**) oleic acid (OL, % in oil), (**viii**) Blackleg (Phoma) disease score (BL), scale from 1 (very susceptible) to 9 (very resistant) and (**ix**) weight of 100 seeds (SW100, g) based on the slope of the linear regression of (**a**) PBV of historical varieties in the field trials over years of variety release from 1996 to 2015 (IP Australia 2022), and (**b**) PBV of the breeding population over cycle 2 (2016 C2), cycle 3 (2018 C3) and cycle 4 (2020 C4) showing box plots of PBV of candidates in each cycle. PBV were obtained from the optimum MMM-FA model of the genotype×environment interaction effects (see Table 2). Data from cycle 1 (2014 C1) were excluded from the analysis and the predicted value for cycle 5 (2015 C5) is from OCS, so C1 and C5 are shown in parentheses. Historical varieties with abbreviations and release dates were Karoo (K, 1996), ATR Beacon (B, 2002), ATR Stubby (Sb, 2003), Bravo TT (Br, 2004), Tornado TT (T, 2004), Banjo TT (Ba, 2005), Crusher TT (C, 2010), ATR Gem (G, 2011), ATR Stingray (Sy, 2011), Sturt TT (St, 2012), ATR Wahoo (W, 2013), ATR Bonito (Bo, 2013), and ATR Mako (M, 2015). Significance of the slope of the linear regressions: * P < 0.05, ** P < 0.01, *** P < 0.001, NS not significant.

(i) Economic index

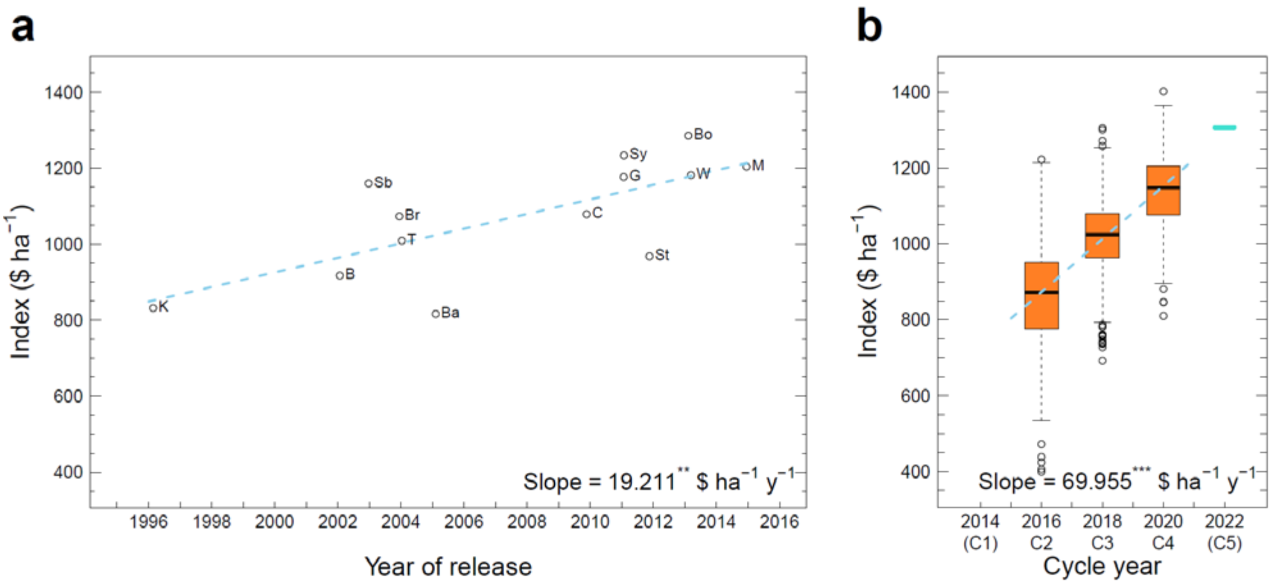

(ii) Days to 50% flower (DTF, days)

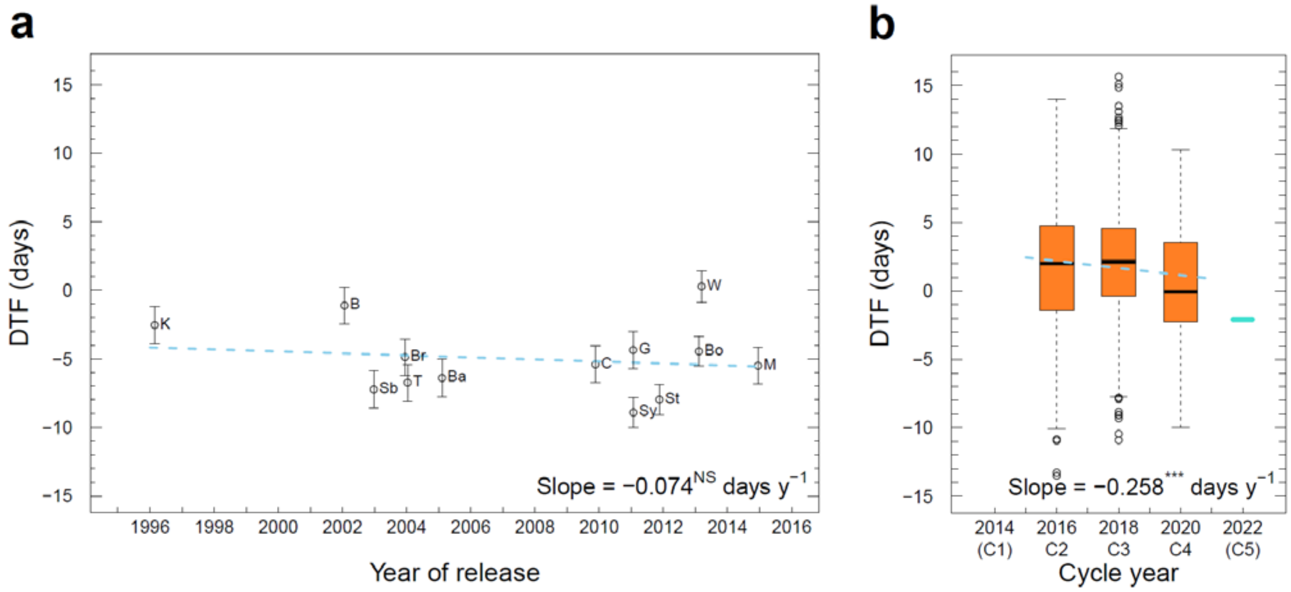

(iii) Plant height (PlHt, cm)

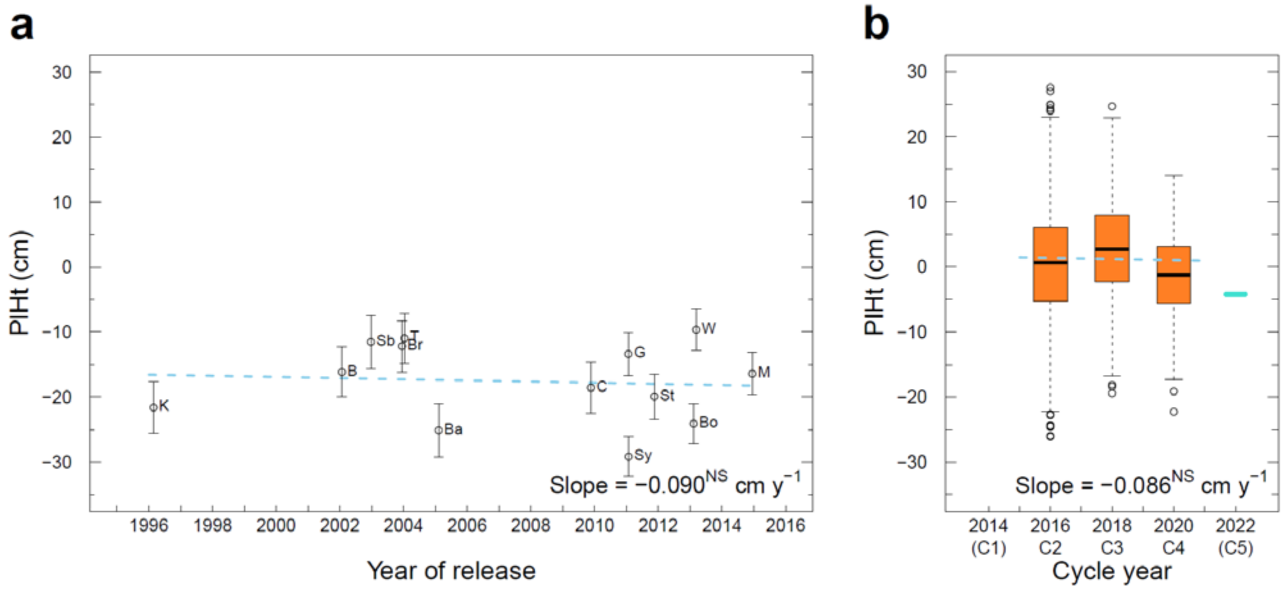

(iv) Seed oil (Oil, %) at 6% moisture

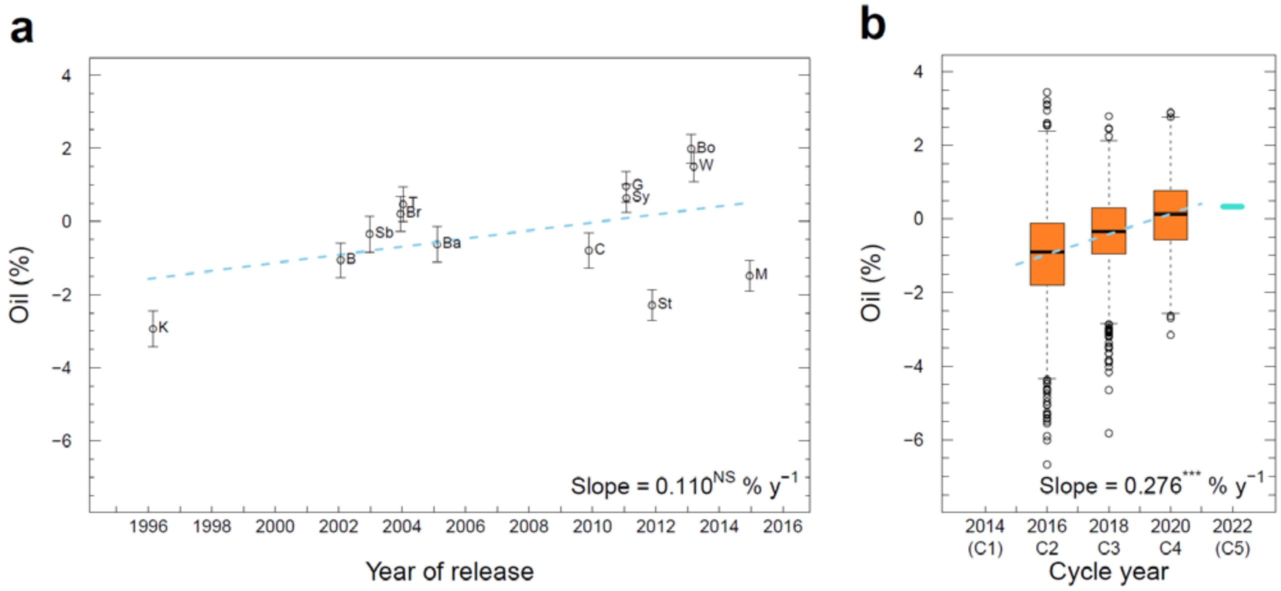

(v) Protein in Meal (ProM, %) at 10% moisture

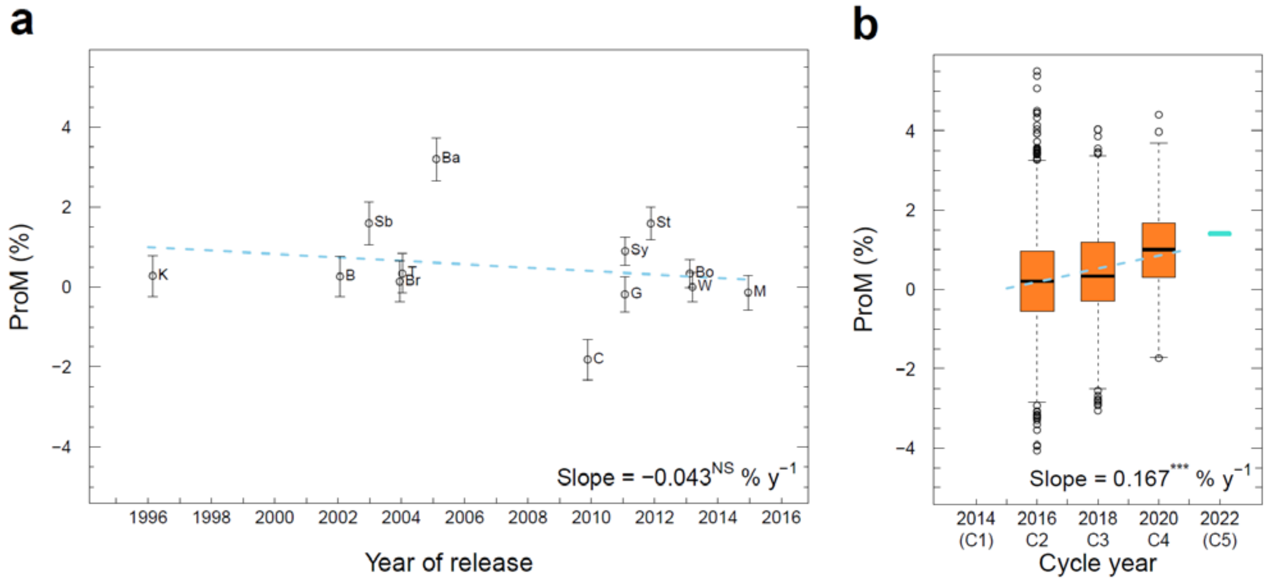

(vi) Glucosinolates in seed (GSL, µmole g^{-1})

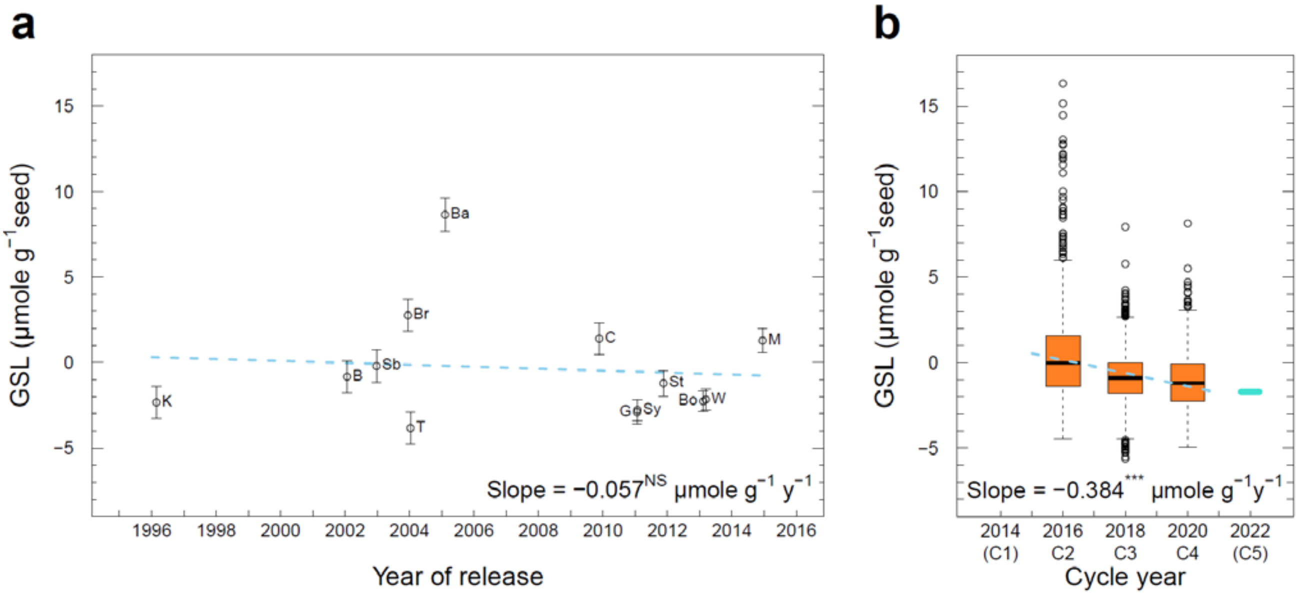

(vii) Oleic acid (OL, % in oil)

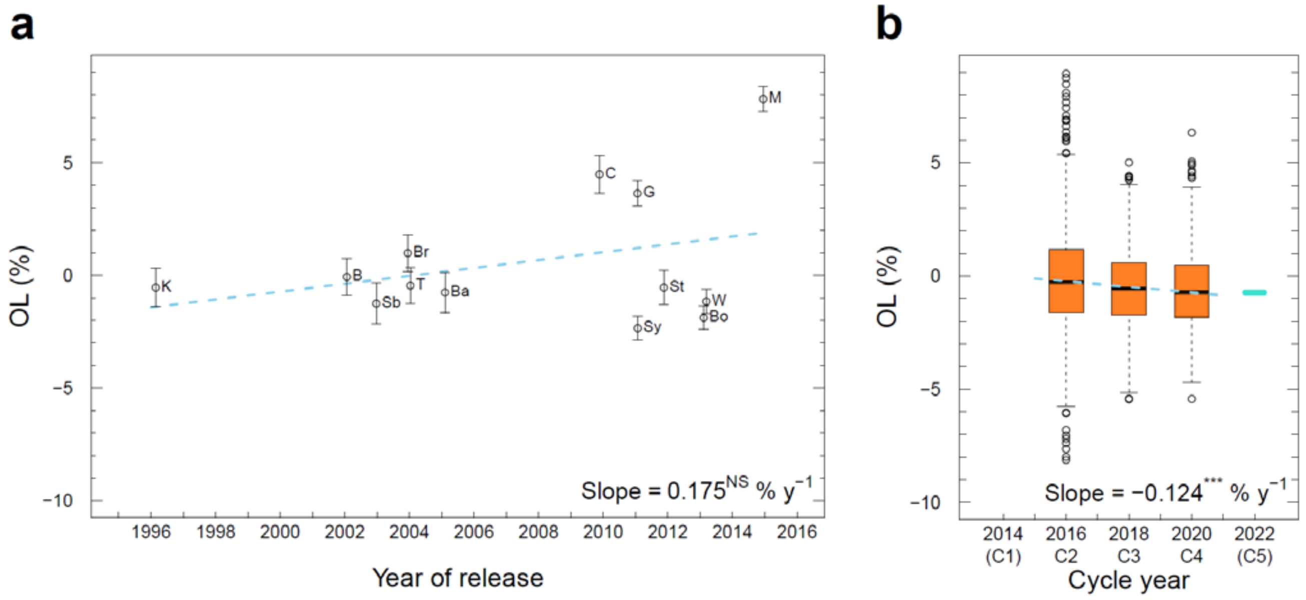

(viii) Blackleg (Phoma) disease score (BL), scale from 1 (very susceptible) to 9 (very resistant)

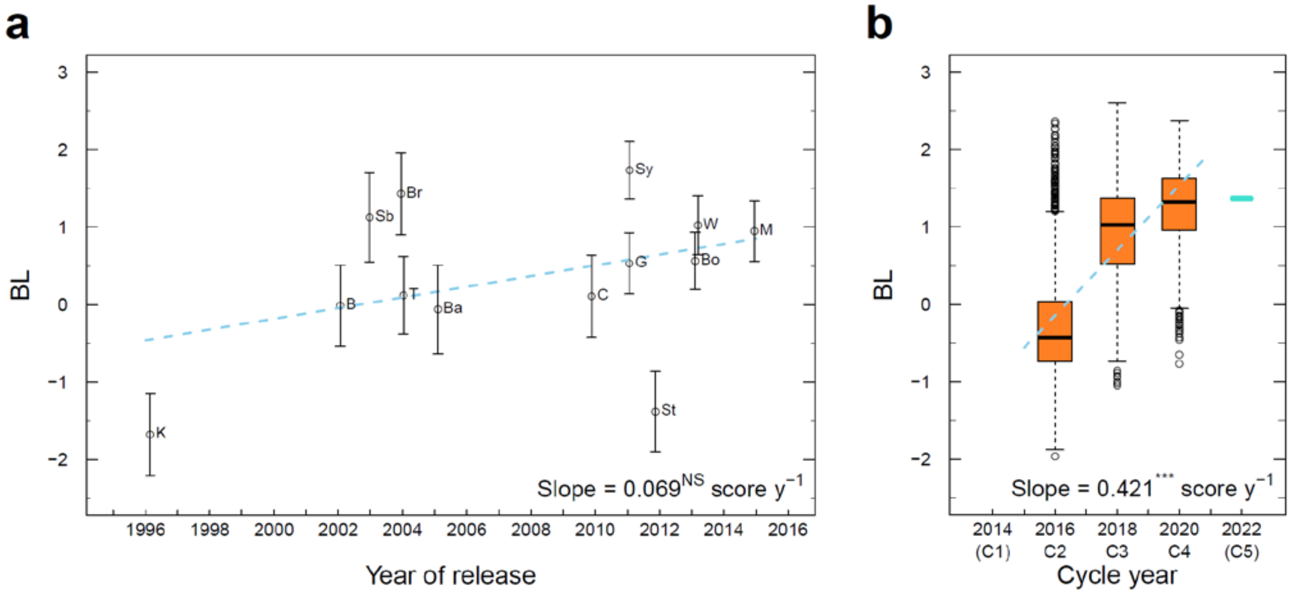

(ix) Weight of 100 seeds (SW100, g)

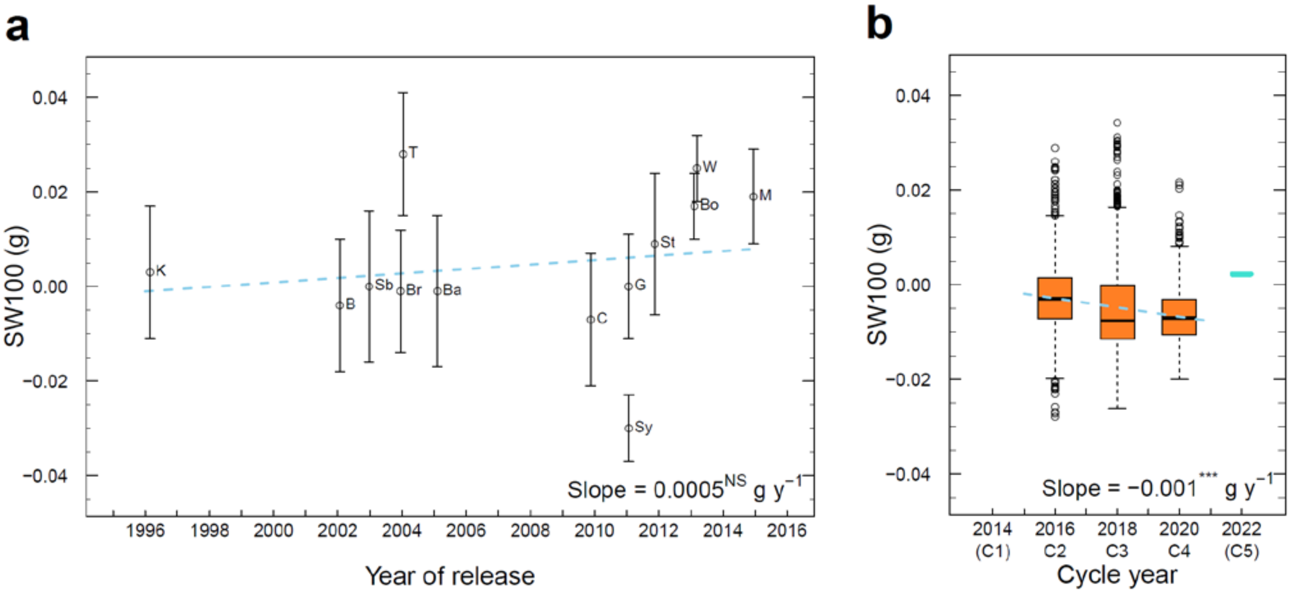

Supplement: Supplementary file 1 [file plants-12-00383-s001.zip › plants-2061854-supplemental files/Online Resource S10_genetic gain PBV all traits_submit.pdf]
