# Supplementary material for: Optimal Contribution Selection Improves the Rate of Genetic Gain in Grain Yield and Yield Stability in Spring Canola in Australia and Canada
Source: Plants (Basel). 2023 Jan 13;12(2):383. doi: 10.3390/plants12020383 (PMC9863350; doi:10.3390/plants12020383)

Rapid and stable global genetic gain in crop breeding: MDPI Plants
Wallace A Cowling*, Felipe Castro-Urrea, Katia T Stefanova, Li Li, Robert G Banks, Renu Saradadevi, Olaf Sass, Brian P Kinghorn & Kadambot H M Siddique
*Corresponding author, The University of Western Australia, wallace.cowling@uwa.edu.au

**Online Resource S2.** Flow of germplasm during four two-year cycles of augmented $S_{0,1}$ family selection in spring canola, based on 64 elite founder lines of which 32 were from the northern hemisphere in Canada (CA) or Europe (EU) and 32 from the southern hemisphere in Australia (AU). Founder lines were intercrossed CA×AU and EU×AU and their $F_1$ progeny were intercrossed to begin field testing of $S_{0,1}$ families in cycle 1 in 2014. Recurrent selection cycles continued every two years with 70 new migrants from CA, EU and AU entering the breeding program in cycles 2, 3 and 4 as $F_1$ progeny CA×AU or EU×AU.

(a) Founders and migrants, and number of crosses between cycles. Crosses for cycles 1 and 2 were designed by the breeder to mix the pedigrees, and crosses for cycles 3, 4 and 5 were designed by optimal contributions selection (OCS).

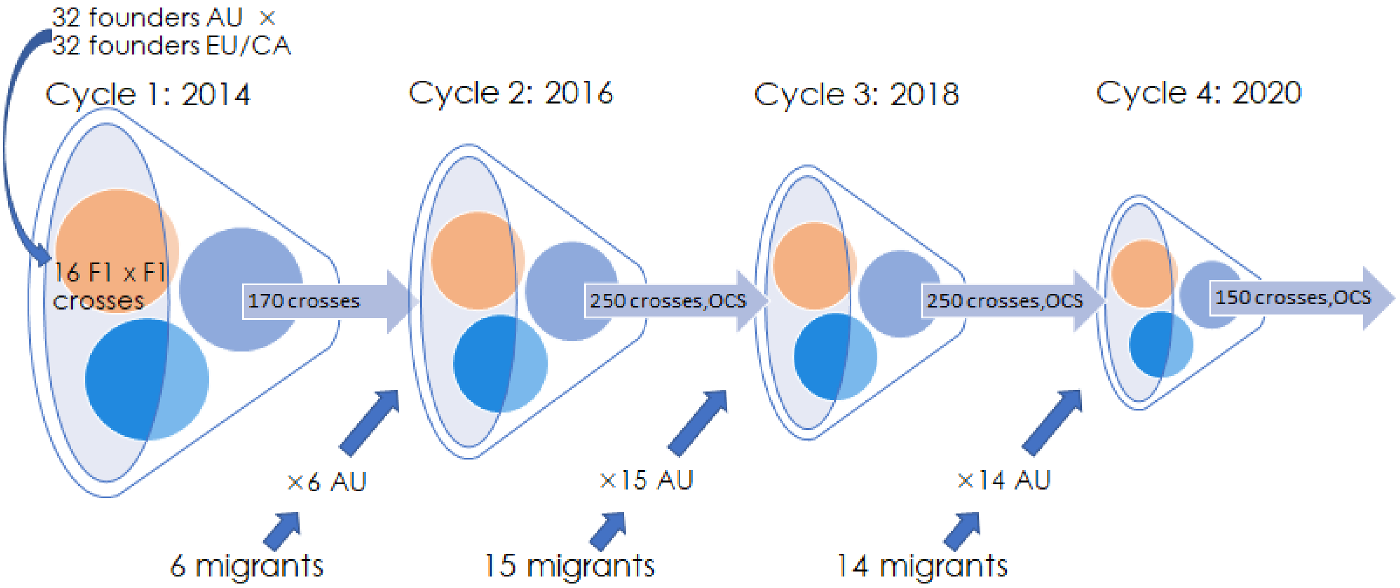


(b) Flow of germplasm showing trial sites in each cycle.

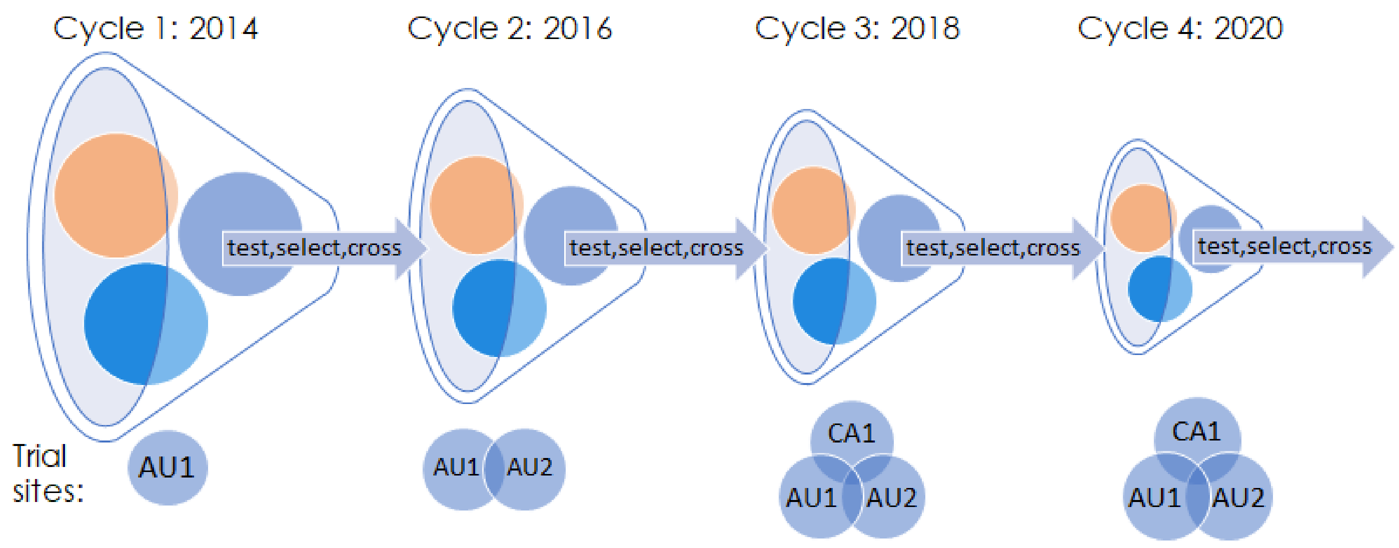

Supplement: Supplementary file 1 [file plants-12-00383-s001.zip › plants-2061854-supplemental files/Online Resource S2_Flow of germplasm cycles_submit.pdf]
