# Supplementary material for: Optimal Contribution Selection Improves the Rate of Genetic Gain in Grain Yield and Yield Stability in Spring Canola in Australia and Canada
Source: Plants (Basel). 2023 Jan 13;12(2):383. doi: 10.3390/plants12020383 (PMC9863350; doi:10.3390/plants12020383)
Supplement: Supplementary file 1 [file plants-12-00383-s001.zip › plants-2061854-supplemental files/Online Resource S5_Statistical model description_submit.pdf]

Rapid and stable global genetic gain in crop breeding: MDPI Plants

Wallace A Cowling*, Felipe Castro-Urrea, Katia T Stefanova, Li Li, Robert G Banks, Renu Saradadevi, Olaf Sass, Brian P Kinghorn & Kadambot H M Siddique

*Corresponding author, The University of Western Australia, wallace.cowling@uwa.edu.au

Online Resource S5. Statistical models description.

Statistical models were adapted from Beeck et al. [6], Cullis et al.[16], Kelly et al. [28], Oakey et al. [35], Smith Alison et al. [52] and Stefanova and Buirchell [53].

Preliminary single site analysis

In the model with pedigree information, let $\boldsymbol{u_g}$ represent the vector of genotype entries in a trial and $\boldsymbol{u_g} = \boldsymbol{u_a} + \boldsymbol{u_{\bar{a}}}$, with $\boldsymbol{u_a}$ representing the vector of trial entries additive genetic effects and $\boldsymbol{u_{\bar{a}}}$ the vector of trial entries non-additive genetic effects; the preliminary single-site model for the observations vector $\boldsymbol{y_j}$ for the *j*th trial (*j* = 1…*t,* with *t* being the number of trials) can be written as

$$\boldsymbol{y_j = X_j\beta_j + Z_{g_j}u_{a_j} + Z_{g_j}u_{\bar{a}_j} + Z_{p_j}u_{p_j} + e_j} \qquad [\mathbf{1}]$$

where, $\boldsymbol{\beta_j}$, $\boldsymbol{u_{a_j}}$, $\boldsymbol{u_{\bar{a}_j}}$, $\boldsymbol{u_{p_j}}$ correspond to fixed effects, random additive genetic effects, random non-additive genetic effects, and random non-genetic effects, respectively, with their design matrices $\boldsymbol{X_j}$, $\boldsymbol{Z_{g_j}}$, $\boldsymbol{Z_{p_j}}$. Plot residuals effects are represented by $\boldsymbol{e_j}$. Random effects are assumed to follow a Gaussian distribution with mean zero and are assumed pairwise independent from each other. The variance models used in random and residual effects are defined as

$$\mathrm{var}(\boldsymbol{u_{a_j}}) = \boldsymbol{A} \otimes \boldsymbol{G_g}$$

$$\mathrm{var}(\boldsymbol{u_{\bar{a}_j}}) = \boldsymbol{I} \otimes \boldsymbol{G_g}$$

$$\mathrm{var}(\boldsymbol{u_{p_j}}) = \boldsymbol{G_{p_j}}$$

$$\mathrm{var}(\boldsymbol{e_j}) = \boldsymbol{R_j}$$

where $\boldsymbol{A}$ is the pedigree relationship matrix, $\boldsymbol{I}$ is an identity matrix and $\boldsymbol{G_g}$, $\boldsymbol{G_p}$ and $\boldsymbol{R}$ correspond to variance matrices.

Base across-sites model

The first across-sites model can be represented as an extension of the model presented in eq. 1. If $\boldsymbol{y}$ represents the concatenated vector of observations for all trials, the model for $\boldsymbol{y}$ can be written as

$$\boldsymbol{y = X\beta + Z_g u_a + Z_g u_{\bar{a}} + Z_p u_p + e} \qquad [2]$$

where terms are as in eq. 1 but now represent concatenated vectors for different trials so that, $\boldsymbol{\beta}$ includes an overall mean of each trial, trial specific modelling terms and dummy variables for missing values in different trials, $\boldsymbol{u_a}$ and $\boldsymbol{u_{\bar{a}}}$ are the vectors of random additive and non-additive genetic effects in each trial, $\boldsymbol{u_p}$ includes non-genetic effects specific to each trial and $\boldsymbol{e}$ is the vector of

residuals partitioned within trials. Random effects follow the same distribution, mean and variances as in the previous model.

Factor analytic modelling across sites

The final across-sites model assumed a factor analytic model by adopting a different form for $\boldsymbol{G_g}$, which resulted in a modified variance structure for $\boldsymbol{u_a}$ and $\boldsymbol{u_{\bar{a}}}$. The model for $\boldsymbol{y}$ is written in the same form as eq. 2, and possesses identical terms and assumptions for its terms except for

$$\mathrm{var}(\boldsymbol{u_a}) = \boldsymbol{A} \otimes \; \boldsymbol{G_g^*}$$

$$\mathrm{var}(\boldsymbol{u_{\bar{a}}}) = \boldsymbol{I} \otimes \; \boldsymbol{G_g^*}$$

$$\boldsymbol{G_g^*} = \; \boldsymbol{\Lambda\Lambda}' + \boldsymbol{\Psi}$$

where $\boldsymbol{\Lambda}$ is a matrix of environmental loadings of dimensions $t \times k$, $\boldsymbol{\Psi}$ is a diagonal matrix of trial specific variances with dimensions $t \times t$, and k is the order of the factor analytic model.
