# Supplementary material for: Optimal Contribution Selection Improves the Rate of Genetic Gain in Grain Yield and Yield Stability in Spring Canola in Australia and Canada
Source: Plants (Basel). 2023 Jan 13;12(2):383. doi: 10.3390/plants12020383 (PMC9863350; doi:10.3390/plants12020383)
Supplement: Supplementary file 1 [file plants-12-00383-s001.zip › plants-2061854-supplemental files/Online Resource S8_Environmental trends all traits sites_submit.pdf]

Rapid and stable global genetic gain in crop breeding: MDPI Plants

Wallace A Cowling*, Felipe Castro-Urrea, Katia T Stefanova, Li Li, Robert G Banks, Renu Saradadevi, Olaf Sass, Brian P Kinghorn & Kadambot H M Siddique
*Corresponding author, The University of Western Australia, wallace.cowling@uwa.edu.au

Online Resource S8. Environmental trends in site predicted means for traits ± standard error across field trials in Australia and Canada in cycle 2 (C2 2016), cycle 3 (C3 2018) and cycle 4 (C4 2020) from the optimum multiplicative mixed model with factor analytic modelling of the genotype by environment effects (see Table 3). Traits include (**a**) days to 50% flower (DTF), (**b**) plant height (PlHt, cm), (**c**) seed oil (Oil, %), (**d**) protein in meal (ProM, %), (**e**) seed glucosinolates (GSL, µmole g^{-1} seed), (**f**) oleic acid (OL, %), (**g**) Phoma (blackleg) resistance score (BL, units from 1-9), and (**h**) weight of 100 seeds (SW100, g). For description of trial codes, see Table 1. The slope of the linear regression of predicted site mean GY across cycles was not significant (NS) or significant at $P < 0.05$ (*).

(a)

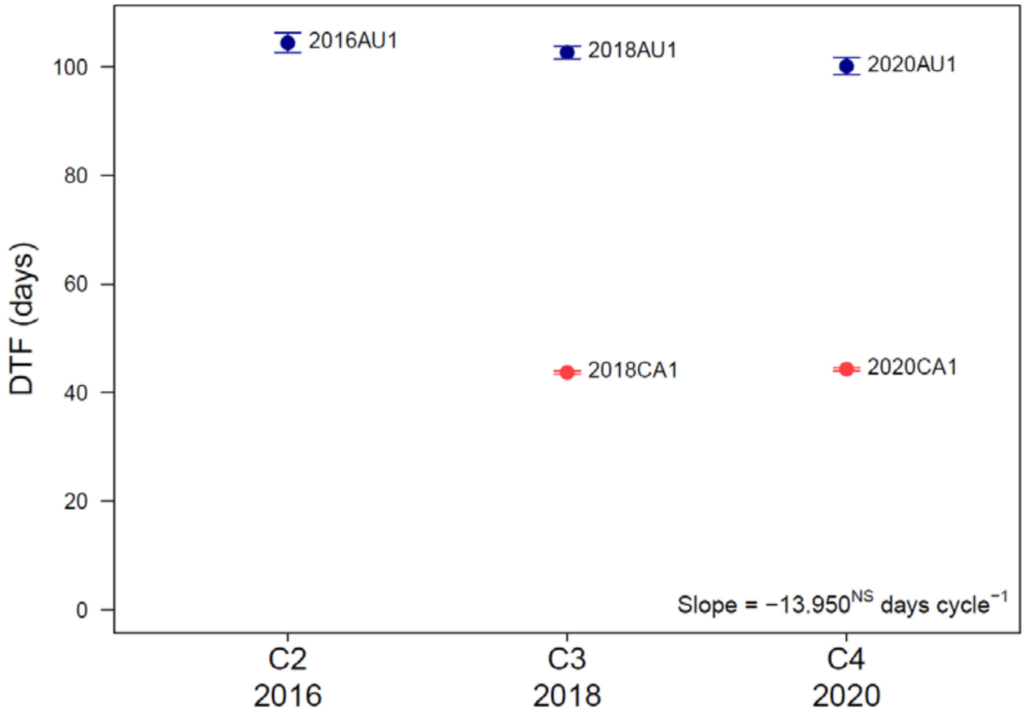

(b)

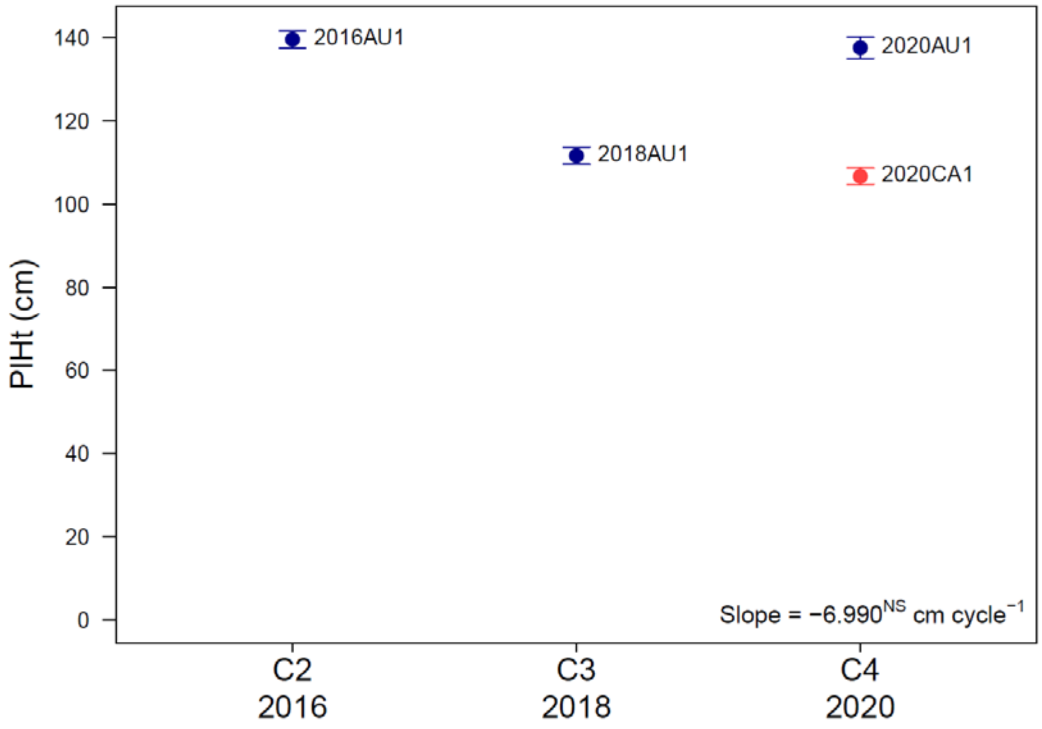

PlHt (cm)
140
120
100
80
60
40
20
0
2016AU1
2018AU1
2020AU1
2020CA1
Slope = −6.990^NS cm cycle^−1
C2
2016
C3
2018
C4
2020

(c)

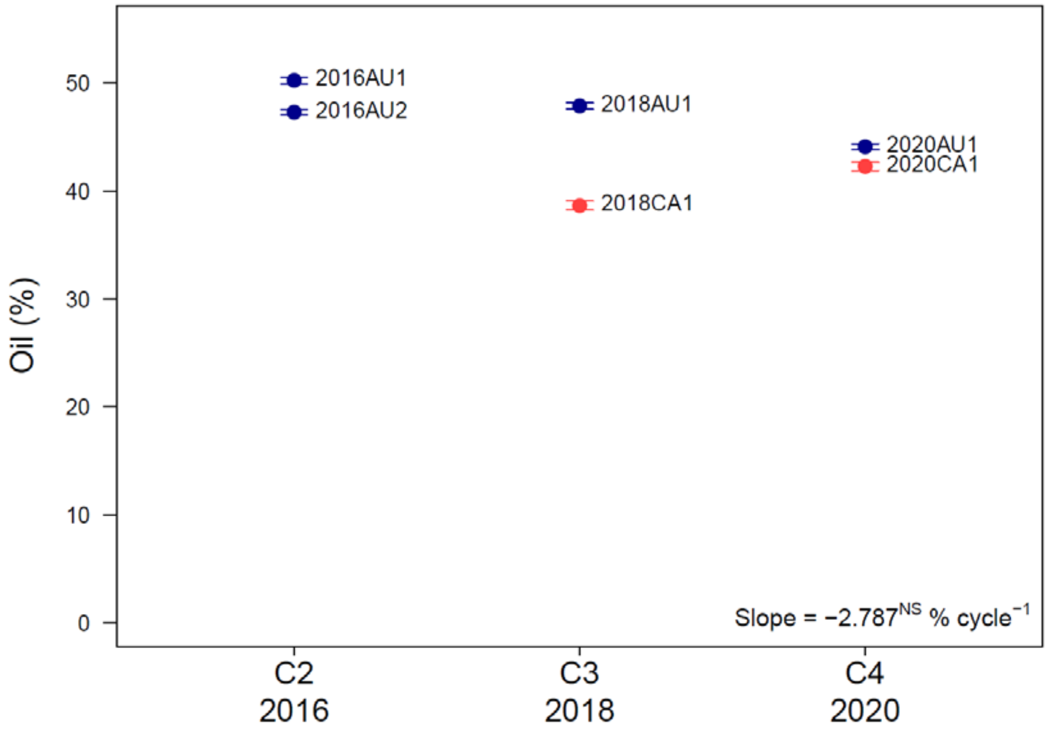

Oil (%)
50
40
30
20
10
0
2016AU1
2016AU2
2018AU1
2018CA1
2020AU1
2020CA1
Slope = −2.787^NS % cycle^−1
C2
2016
C3
2018
C4
2020

(d)

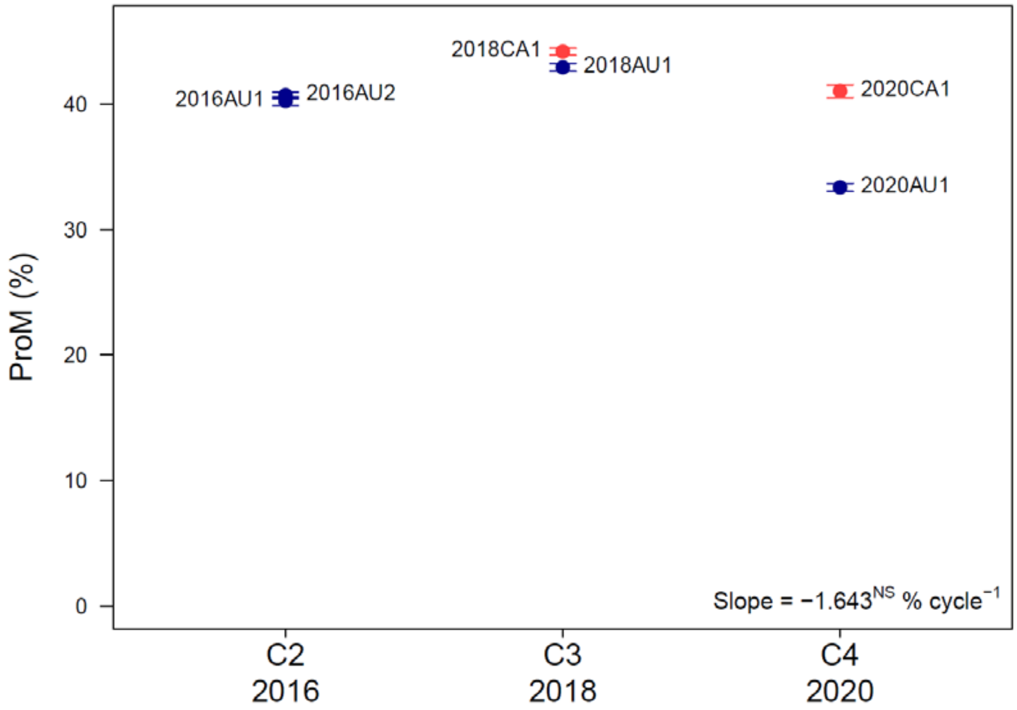
ProM (%)
0
10
20
30
40
2016AU1
2016AU2
2018CA1
2018AU1
2020CA1
2020AU1
Slope = −1.643NS % cycle−1
C2
2016
C3
2018
C4
2020

(e)

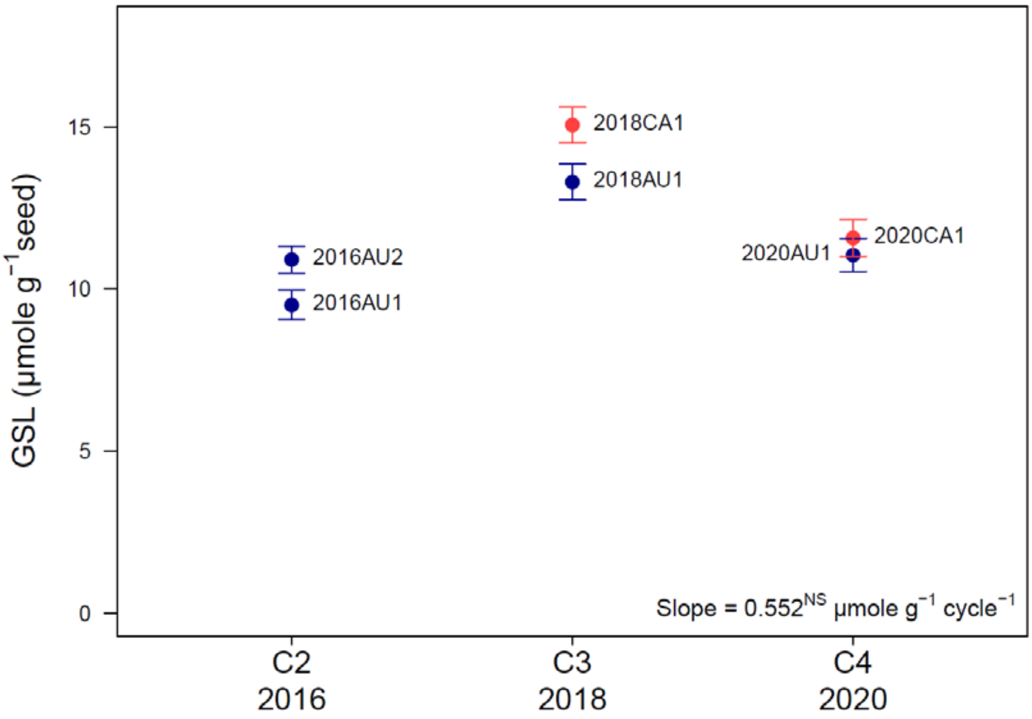
GSL (µmole g−1seed)
0
5
10
15
2016AU2
2016AU1
2018CA1
2018AU1
2020CA1
2020AU1
Slope = 0.552NS µmole g−1 cycle−1
C2
2016
C3
2018
C4
2020

(f)

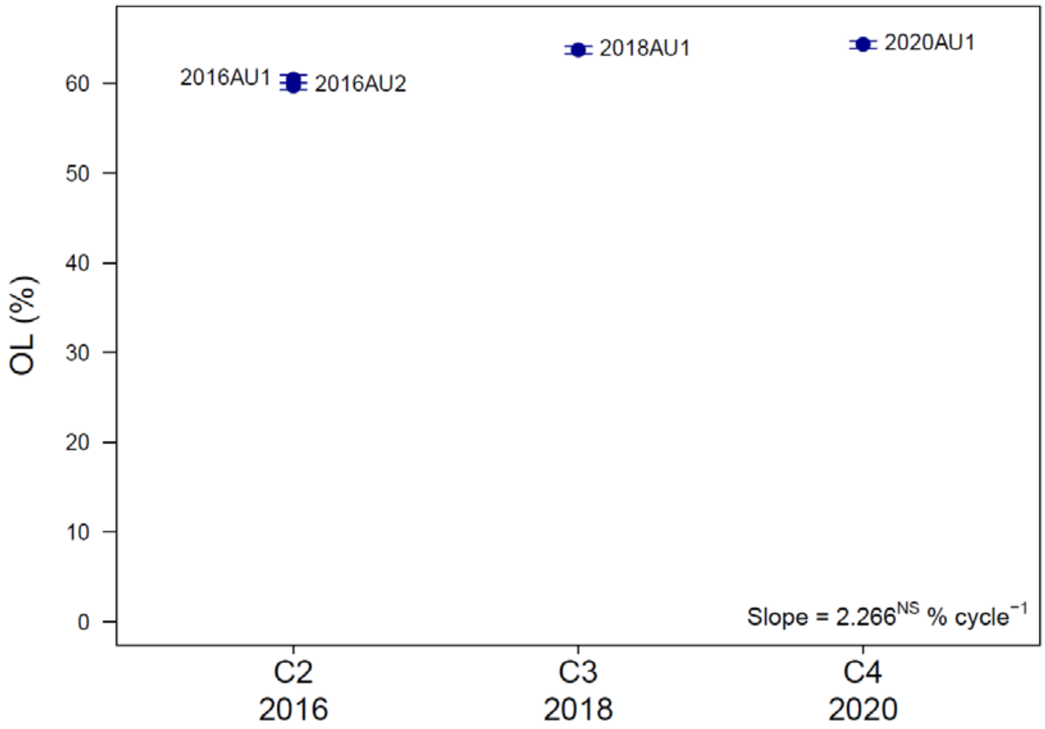

OL (%)
0
10
20
30
40
50
60
2016AU1
2016AU2
2018AU1
2020AU1
Slope = 2.266^{NS} % cycle^{-1}
C2 2016
C3 2018
C4 2020

(g)

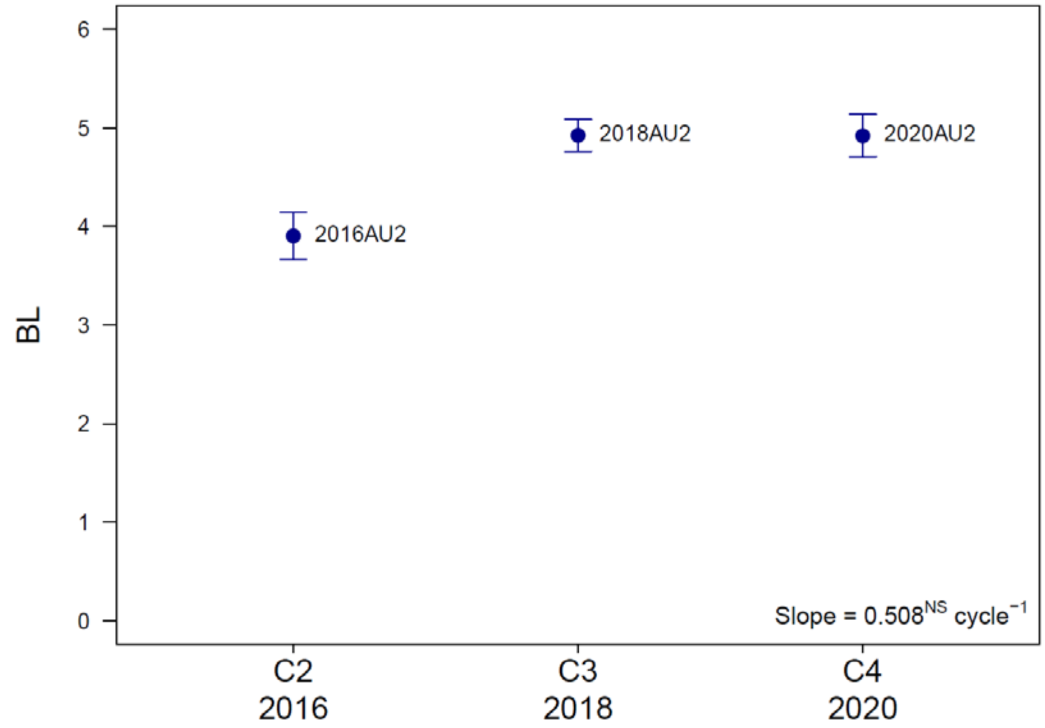

BL
0
1
2
3
4
5
6
2016AU2
2018AU2
2020AU2
Slope = 0.508^{NS} cycle^{-1}
C2 2016
C3 2018
C4 2020

(h)

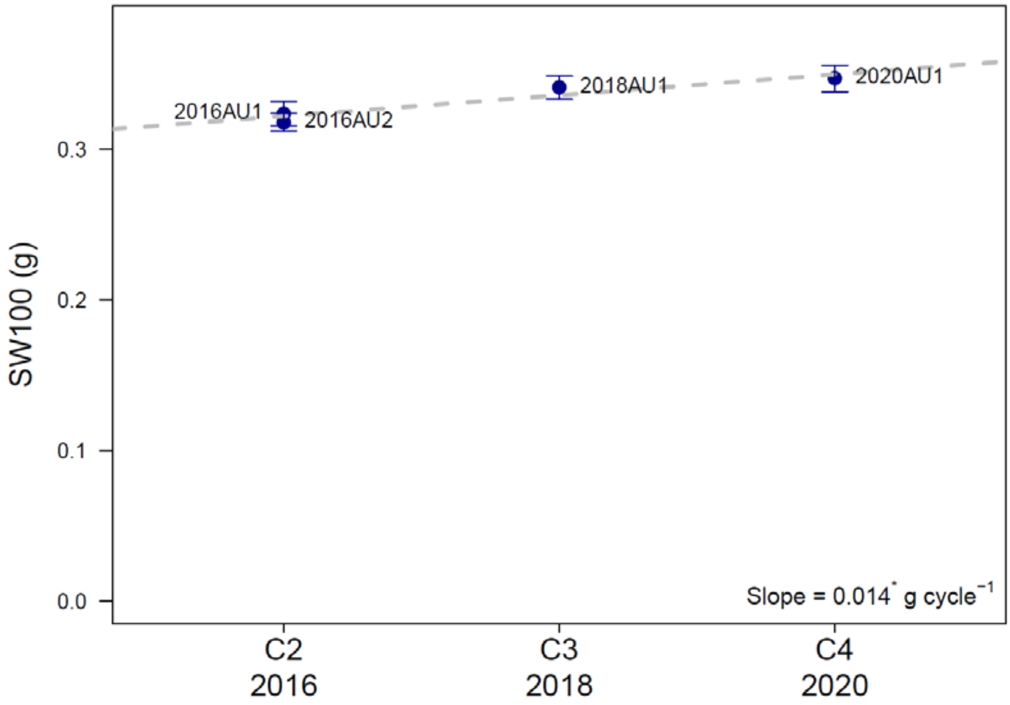

SW100 (g)
0.0
0.1
0.2
0.3
2016AU1
2016AU2
2018AU1
2020AU1
Slope = 0.014* g cycle−1
C2
2016
C3
2018
C4
2020
